# A heterobimetallic complex featuring a Ti–Co multiple bond and its application to the reductive coupling of ketones to alkenes†
†Electronic supplementary information (ESI) available: Experimental procedures, additional spectroscopic data for **1–4**, and computational details of **2** and **3**. CCDC 1037714–1037716. For ESI and crystallographic data in CIF or other electronic format see DOI: 10.1039/c4sc03772c



**DOI:** 10.1039/c4sc03772c

**Published:** 2015-01-19

**Authors:** Bing Wu, Mark W. Bezpalko, Bruce M. Foxman, Christine M. Thomas

**Affiliations:** a Department of Chemistry , Brandeis University , 415 South Street MS 015 , Waltham , MA 02454 , USA . Email: thomasc@brandeis.edu

## Abstract

To explore metal–metal multiple bonds between first row transition metals, Ti/Co complexes supported by two phosphinoamide ligands have been synthesized and characterized. The Ti metalloligand Cl_2_Ti(XylNP^i^Pr_2_)_2_ (**1**) was treated with CoI_2_ under reducing conditions, permitting isolation of the Ti/Co complex [(μ-Cl)Ti(XylNP^i^Pr_2_)_2_CoI]_2_ (**2**). One electron reduction of complex **2** affords ClTi(XylNP^i^Pr_2_)_2_CoPMe_3_ (**3**), which features a metal–metal triple bond and an unprecedentedly short Ti–Co distance of 2.0236(9) Å. This complex is shown to promote the McMurry coupling reaction of aryl ketones into alkenes, with concomitant formation of the tetranuclear complex [Ti(μ_3_-O)(NXylP^i^Pr_2_)_2_CoI]_2_ (**4**). A cooperative mechanism involving bimetallic C=O bond activation and a cobalt carbene intermediate is proposed.

## Abstract 2

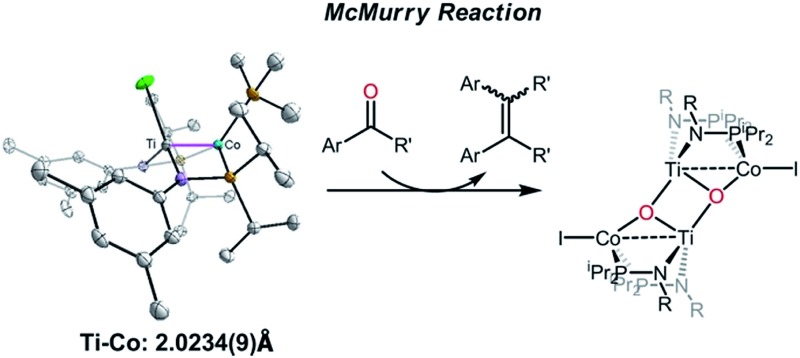
A Ti/Co heterobimetallic complex featuring a very short metal–metal triple bond has been synthesized. This complex promotes the reductive coupling reaction of aryl ketones into alkenes.

## Introduction

Metal–metal interactions have received considerable attention owing to their fundamentally interesting bonding properties and their potential use in multi-electron transfer processes.^1–8^ In particular, interactions between early and late metals are of interest due to their relevance to observed enhanced reactivity phenomena in the area of surface catalysis, *e.g.* the Fischer–Tropsch process.^9,10^ The formation of “strong metal-support interactions” in heterogeneous catalytic systems involving early metal-oxide supported late metal catalysts has been shown to promote reactivity,^11–13^ with the reduction of the early metal and formation of direct early/late metal–metal interactions being observed. To emulate this behavior in homogeneous systems, some metal–metal bonded early/late heterobimetallic complexes have been reported over the past several decades, and their reactivity towards small molecule substrates has been explored.^3,5–8,14^

**Scheme 1:**
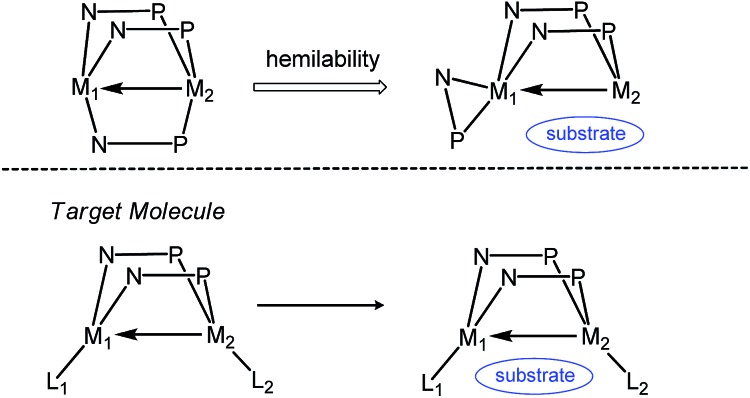
Justification for exploring a bis(phosphinoamide) ligand framework for heterobimetallic complexes.

Wolczanski's report of a metal–metal triple bond in the Ti/Rh complex Ti(OCMe_2_CH_2_PPh_2_)_3_Rh represents an important breakthrough in the area of early/late heterobimetallic complexes.^15^ Our group has studied a similar series of *C*_3_-symmetric tris(phosphinoamide) Zr–Co heterobimetallic complexes featuring Zr–Co multiple bonds,^16–18^ and their remarkable ability to undergo a wide array of one, two, and four-electron transformations with small molecules such as hydrazines,^19^ alkyl halides,^20,21^ CO_2_,^22^ diaryl ketones,^23–25^ and organic azides.^26^ Since the dissociation of one of the phosphine ligands from Co and its coordination to Zr in an η^2^ fashion are involved in many of our examples of reactivity,^20,22,24–26^ we posited that coordinatively unsaturated heterobimetallic complexes linked by just two phosphinoamide ligands may lead to more reactive and catalytically competent compounds (Scheme 1). Such a strategy was employed in the “A frame” heterobimetallic bis(alkoxyphosphine)-linked Zr/M (M = Rh, Pt) complexes reported by Wolczanski *et al.*, permitting the observation of intermetallic alkyl group exchange.^27–29^ However, other interesting cooperative reactivity was likely hindered in these systems by the coordinative saturation provided by a Cp* ligand on Zr.

Furthermore, in light of the wide use of TiO_2_ as a catalyst support in heterogeneous catalysis and titanium's ability to undergo one-electron redox processes, we chose to extend our studies of heterobimetallic chemistry to Ti/M complexes. Nagashima and co-workers have reported a series of bis(phosphinoamide) Ti^IV^/M (M = Ni, Pd, Pt, and Ru) heterobimetallic complexes, however, the reported reactivity of these complexes remains somewhat limited.^30–32^ Herein, we describe a synthetic method to construct metal–metal multiple bonds between Ti and a redox active first row transition metal, Co. Further, we explore the reactivity of a Ti/Co complex with aryl ketones, uncovering that the two metal centers in this heterobimetallic complex mediate the reductive coupling of ketones to alkenes (the McMurry reaction). Other reported examples of the deoxygenation of ketones by an early/late heterobimetallic complex include Gade's report of the transfer of an oxygen atom from cyclopropenone to a bound carbonyl ligand by HC(Me_2_SiN(2,3,4-F_3_C_6_H_2_))_3_Zr–Fe(CO)_2_Cp,^33,34^ and our own report of cleavage of the C=O bond of benzophenone by (THF)Zr(MesNP^i^Pr_2_)_3_CoN_2_ (Mes = 2,4,6-trimethylphenyl) to generate the μ-oxo/carbene complex (η^2^-MesNP^i^Pr_2_)Zr(μ-O)(MesNP^i^Pr_2_)_2_Co=CPh_2_.^24^

## Results and discussion

**Scheme 2:**
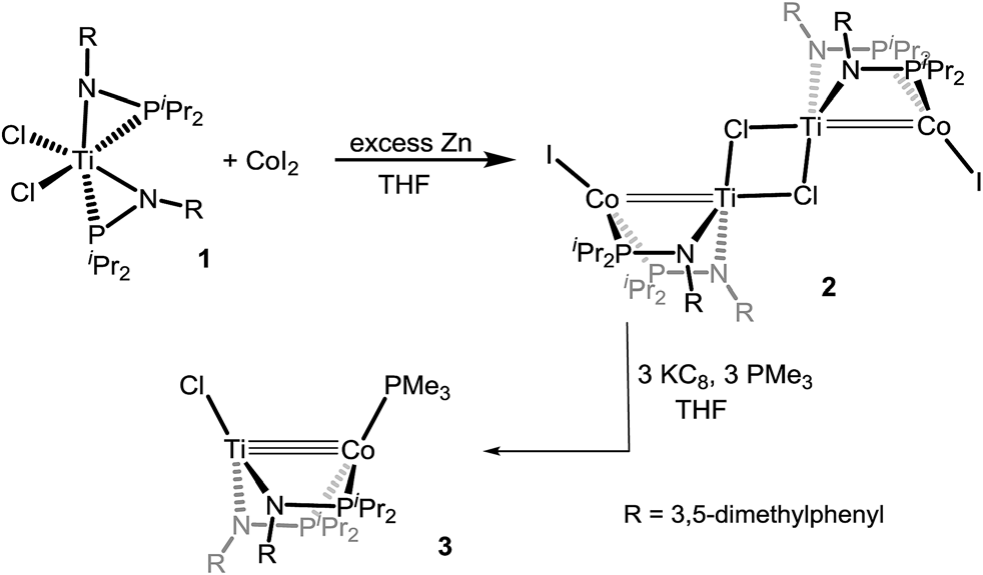
Synthesis of heterobimetallic Ti–Co complexes.

Despite our previous successes with heterobimetallic Zr/Co complexes featuring the [MesNP^i^Pr_2_]^–^ ligand,^16,17^ our initial efforts targeting bimetallic Ti(MesNP^i^Pr_2_)_*n*_Co complexes were largely unsuccessful as a result of the smaller ionic radius of Ti and the resulting inability to install more than one phosphinoamide ligand around the Ti center. We found, however, that reducing the steric hindrance by changing the *N*-aryl group from 2,4,6-trimethylphenyl to 3,5-dimethylphenyl permits access to the desired precursor Cl_2_Ti(XylNP^i^Pr_2_)_2_ (**1**) (Xyl = 3,5-dimethylphenyl) *via* treatment of TiCl_4_ with two equivalents of Li[XylNP^i^Pr_2_] (see ESI†). In contrast to our reported synthesis of ClZr(MesN^i^Pr_2_)_3_CoI,^16^ successful installation of a second metal to metalloligand **1** required the addition of an external reductant. Treatment of CoI_2_ with **1** in the presence of excess Zn powder afforded a symmetric tetrametallic complex [(μ-Cl)Ti(XylNP^i^Pr_2_)_2_CoI]_2_ (**2**) with two heterobimetallic Ti–Co fragments bridged by two Cl^–^ ligands between the two Ti centers (Scheme 2).

**Fig. 1:**
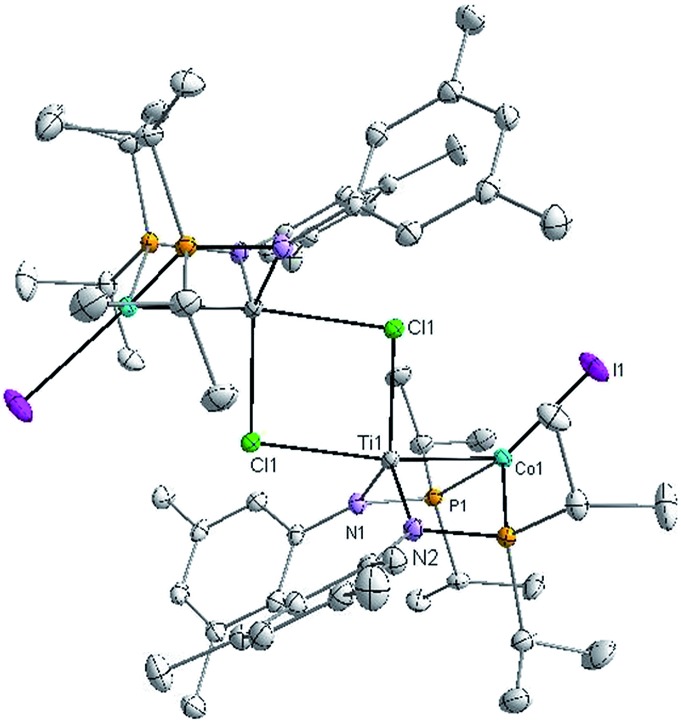
Displacement ellipsoid (50%) representation of **2**. All hydrogen atoms have been omitted for clarity. Selected bond distances (Å) and angles (°): Ti1–Co1, 2.2051(4); Ti1–Cl1, 2.3843(5), 2.5745(5); Cl1–Ti1–N1, 126.73(4); Cl1–Ti1–N2, 115.90(4); N1–Ti1–N2, 116.91(6); P1–Co1–I1, 119.458(14); P2–Co1–I1, 125.626(14); P1–Co1–P2, 111.923(17).

X-ray crystallography of single crystals of **2** reveals a Ti–Co interatomic distance of 2.2051(4) Å with a “formal shortness ratio” (the ratio of the metal–metal interatomic distance to the sum of the single bond atomic radii of the two metal ions, FSR^1^) of only 0.89, suggesting a relatively strong metal–metal interaction between Ti and Co (Fig. 1). Taking this metal–metal bond into consideration, the Ti center adopts a trigonal bipyramidal geometry and the geometry about Co is distorted tetrahedral. The Ti–Cl bond *trans* to the Ti–Co bond is considerably longer than the one *cis* to the Ti–Co bond (2.5745(5) Å *vs.* 2.3843(5) Å), in line with previous reports of polar metal–metal bonds exerting *trans* influences on apical ligands in heterobimetallic complexes.^16,35^

Formally, the oxidation states of the two metals in **2** are Ti^III^ and Co^I^, and solution magnetic moment measurements suggest an *S* = 1 ground state (*μ*_eff_ = 3.11 *μ*_B_). A computational investigation of **2** was carried out using density functional theory (DFT). Although the unmodified ligand set was used in calculation, the cobalt-bound iodide ligand was changed to chloride for computational simplicity. Mulliken population analysis predicts that the unpaired spin density in [(μ-Cl)Ti(XylNP^i^Pr_2_)_2_CoCl]_2_ (**2′**) resides almost exclusively on the Co atoms (Mulliken spin density of 1.11 on each Co atom). Furthermore, an examination of the frontier molecular orbitals of **2** reveals a σ bonding interaction involving the Co and Ti d_*z* ^2^_ orbitals as well as weak π interactions between the d_*xz*_ and d_*yz*_ orbitals of the Ti and Co centers (see ESI, Fig. S7†). As would be expected based on the differences in molecular orbital energies of Ti and Co, the bonding orbitals are considerably polarized and localized more on the Co center. Nonetheless, a reasonable approximation of the electronic structure of **2** involves pairing of electrons between Ti^III^ and Co^I^ through polar covalent bonding, with additional weak Ti → Co π interactions for an overall Ti–Co bond order of ∼2.

**Fig. 2:**
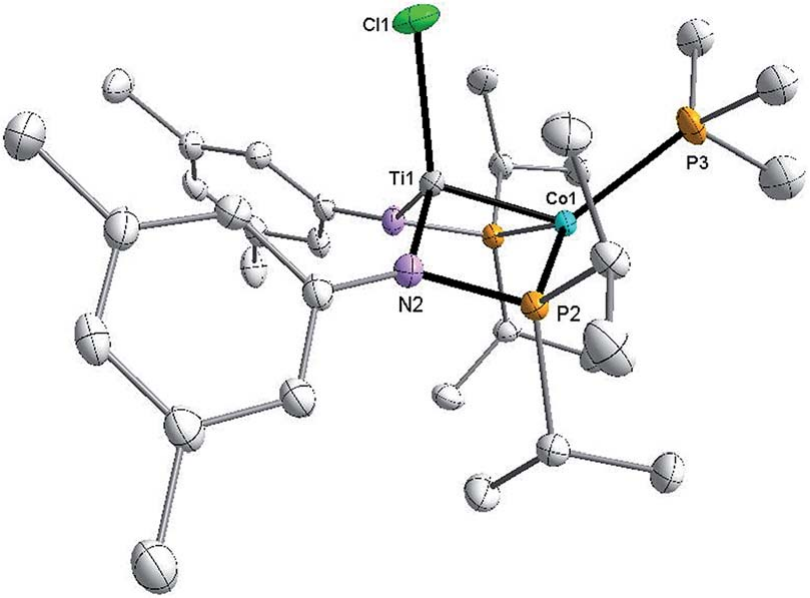
Displacement ellipsoid (50%) representation of **3**. All hydrogen atoms have been omitted for clarity. Selected bond distances (Å) and angles (°): Ti1–Co1, 2.0234(9); Ti1–Cl1, 2.3005(13); Cl1–Ti1–N1, 114.99(14); Cl1–Ti1–N2, 112.73(13); N1–Ti1–N2, 122.00(16); P1–Co1–P3, 118.55(5); P2–Co1–P3, 114.31(5); P1–Co1–P2, 127.13(5).

The reduction of **2** with KC_8_ in the presence of excess PMe_3_ in THF afforded a brown solution from which the diamagnetic heterobimetallic complex ClTi(XylNP^i^Pr_2_)_2_CoPMe_3_ (**3**) could be isolated (Scheme 2). The ^31^P NMR spectrum of complex **3** features two resonances in a 2 : 1 integral ratio at *δ* 44.6 and –21.0, corresponding to the bridging phosphinoamide ligands and Co-bound PMe_3_ ligand, respectively. The structure of complex **3** was determined by single crystal X-ray diffraction, revealing a monomeric heterobimetallic complex (Fig. 2). The decrease in the distance between the two metal centers in **3** in comparison to **2** (2.0236(9) Å *vs.* 2.2051(4) Å, FSR = 0.81 *vs.* 0.89), indicates a substantial increase in Ti–Co bond order upon reduction. Examples of Ti–Co bonds in the literature fall in the range of 2.47–2.61 Å,^36–38^ and the Ti–Co distances in complexes **2** and **3** are, by far, the shortest Ti–Co bonds reported to date. Moreover, the Ti–Co bonds in **2** and **3** represent the shortest bonds ever observed between Ti and any other transition metal, with the closest known analogue being Wolczanski's heterobimetallic Ti/Rh complex Ti(OCMe_2_CH_2_PPh_2_)_3_Rh (Ti–Rh = 2.2142(11) Å, FSR = 0.86).^15^

**Fig. 3:**
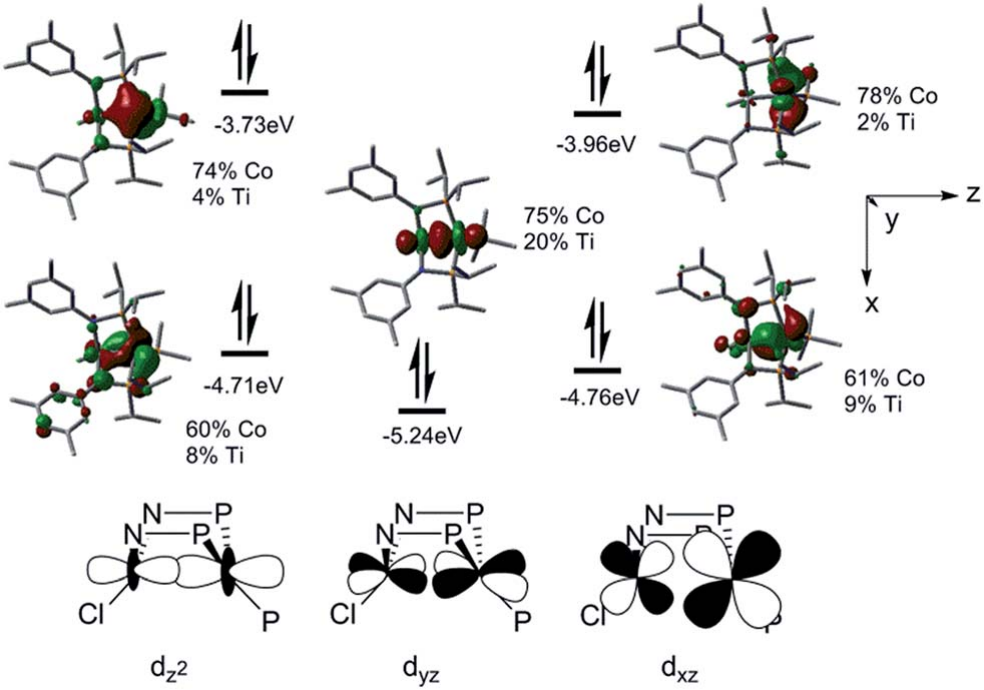
Depictions and energies of the calculated frontier molecular orbitals of **3**.

To better understand the electronic configuration and metal–metal bonding of complex **3**, a computational investigation was undertaken using DFT. Examination of the calculated frontier molecular orbital diagram predicted for complex **3** (Fig. 3) reveals a σ interaction between the Co and Ti d_*z* ^2^_ orbitals, as well as two additional π type interactions involving the Co and Ti d_*xz*_ and d_*yz*_ orbitals. This (σ)^2^(π)^4^(Co_nb_)^4^ configuration gives rise to a bond order of 3, consistent with the short Ti–Co distance. The increased metal–metal bonding in **3** is manifested in a higher calculated Wiberg Bond Index (WBI) between Ti and Co in **3** (WBI = 1.59) compared to **2** (WBI = 1.26). The population of the Kohn–Sham orbitals shown in Fig. 3 are indicative of a polar covalent Ti–Co σ bond with Co and Ti contributing 75% and 20% of the electron density in the bond, respectively. Likewise, a natural bond orbital (NBO) calculation predicts a Ti–Co σ bond with 70.8% Co character and 29.2% Ti character. The two π interactions, on the other hand, are significantly more localized on Co than on Ti, indicating that these are best described as donor–acceptor interactions. The polarity of the Ti–Co bond in **3** is also evident from the difference in natural charges of the two atoms calculated *via* Natural Population Analysis (NPA). As shown in Table 1, the more negative charge on Co and more positive charge on Ti in complex **3** is indicative of a polar bond. The difference in natural charge between the two metal atoms is far smaller for complex **3** (1.04) than for the previously described Zr/Co complex (THF)Zr(MesNP^i^Pr_2_)_3_CoN_2_ (2.61).^39^

**Table 1:** Computed natural charges and Wiberg Bond Indices (WBIs) of Ti/Co complexes **2** and **3**, and Zr/Co complex **6**

	Natural charges		WBI
	Ti	Co	
[(μ-Cl)Ti(XylNP^i^Pr_2_)_2_CoCl]_2_(**2′**)	0.26	–0.25	1.26
ClTi(XylNP^i^Pr_2_)_2_CoPMe_3_(**3**)	0.40	–0.64	1.59
(THF)Zr(MesNP^i^Pr_2_)_3_CoN_2_(**6**)^39^	1.39	–1.22	0.95
(H_2_N)_3_Ti–Co(CO)_4_ (ref. 37)^*a*^	1.25	–0.19	0.30
(H_2_N)_3_Ti–Co(CO)_3_(PH_3_) (ref. 37)^*a*^	1.26	–0.24	0.36

^*a*^Model complexes calculated using the B3LYP functional and TZVDP basis set.

Based on these metrics, the Ti–Co bond is more covalent than the metal–metal bond in the (THF)Zr(MesNP^i^Pr_2_)_3_CoN_2_ system,^39^ likely owing to the better orbital overlap between the two sets of 3d orbitals (rather than 3d and 4d) in the Ti–Co complex. The enhanced orbital overlap between the two first row metals in complex **3** is likely also responsible for its shorter metal–metal distance compared to the isoelectronic complex Ti(OCMe_2_CH_2_PPh_2_)_3_Rh.^15^

The *formal* oxidation states of the two metals in **3** could be described as Ti^III^/Co^0^ or Ti^IV^/Co^–I^. A recent study using X-ray absorption near edge structure (XANES) spectroscopy showed that the effective oxidation states in the similar complex (THF)Zr(MesNP^i^Pr_2_)_3_CoN_2_ are Zr^IV^/Co^–I^.^39^ However, given the polar covalent nature of the metal–metal σ bond, a non-zwitterionic Ti^III^/Co^0^ description may be favored in the case of **3** and further spectroscopic study will be required to determine the effective oxidation states in this molecule.

It is also worth noting that Gade's Ti/Co complexes MeSi(Me_2_SiN(4-CH_3_C_6_H_4_))_3_Ti–Co(CO)_3_L (L = CO, P(Tol_3_)) have much weaker Ti–Co bonds than **2** or **3** based on their longer intermetallic distances (2.5542(10) Å and 2.471(4) Å, respectively).^37^ The metal–metal bonds in these molecules were also deemed polar covalent in nature. The calculated WBIs of simplified models of Gade's complexes are much smaller than those computed for **2** and **3**, and the computed charge differences between Ti and Co are larger (Table 1). The weaker and more polar bonding between Ti and Co in MeSi(Me_2_SiN(4-CH_3_C_6_H_4_))_3_Ti–Co(CO)_3_L may be a function of oxidation state differences: as shown in Table 1, the natural charge calculated for the Ti atom in the (NH_2_)_3_Ti–Co(CO)_3_L complexes is greater than that of **3** by ∼1, implicating a more reduced Ti^III^ center in **2** and **3**.

**Scheme 3:**
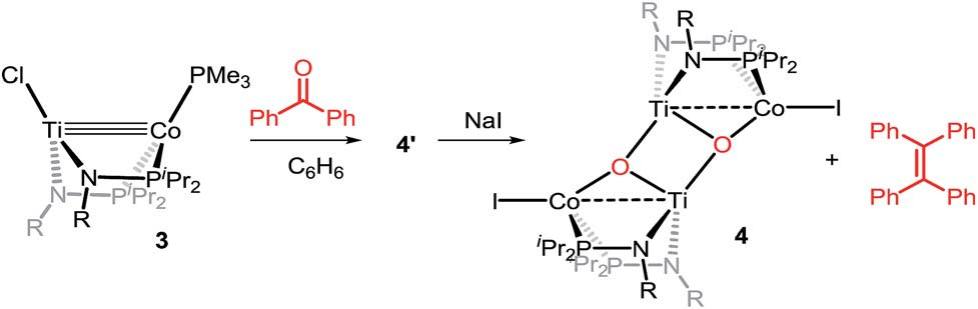
Reactivity of **3** with benzophenone.

**Fig. 4:**
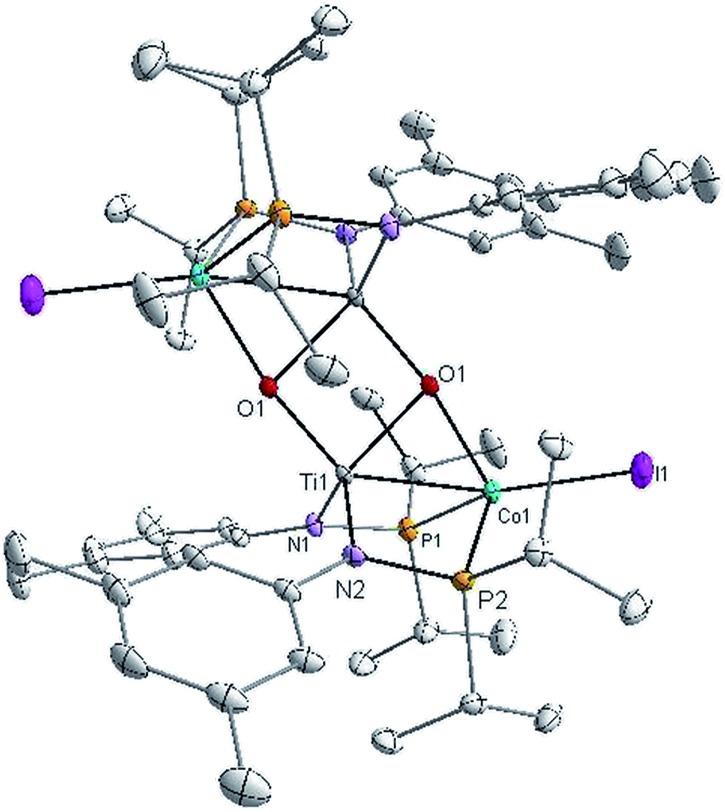
Displacement ellipsoid (50%) representation of **4**. All hydrogen atoms and a disordered benzene solvent molecule have been omitted for clarity. Selected bond distances (Å) and angles (°): Ti1–Co1, 2.4397(5); Ti1–O1, 1.8456(15), 1.9342(15); Co1–O1, 2.0340(14); O1–Ti1–N2, 112.22(7), 106.81(7); O1–Ti1–N1, 112.17(7), 119.27(7); P1–Co1–I1, 117.839(18); P2–Co1–I1, 116.582(18); P1–Co1–O1, 99.65(4); P2–Co1–O1, 96.61(4).

Owing to its open coordination geometry, the bis(phosphinoamide) Ti/Co platform is expected to be able to accommodate a wide range of small molecule substrates in the context of σ and π bond activation, and we chose to initially investigate the representative reactivity of **3** with diaryl ketones. Allowing a benzene solution of **3** to react with benzophenone at room temperature resulted in the loss of PMe_3_ (detected by ^31^P NMR spectroscopy) and afforded a brown complex **4′** that featured paramagnetically shifted resonances in its ^1^H NMR spectrum (Scheme 3, Fig. S5†). Several attempts to crystallize this complex failed due to its thermal instability. However, addition of NaI to a solution of **4′** generated a purple Ti^IV^Co^I^ complex [Ti(μ_3_-O)(XylNP^i^Pr_2_)_2_CoI]_2_ (**4**) along with tetraphenylethylene, which was observed by GC-MS (*m*/*z* = 332). The structure of **4** was determined by a single-crystal X-ray diffraction study, revealing a tetrametallic structure in which a μ_3_-oxo ligand bridges between Ti and Co as well as a second Ti center, giving a dimeric structure (Fig. 4). The tetrametallic complex **4** features an elongated Ti–Co distance of 2.4397(4) Å (FSR = 0.985), indicating a diminished metal–metal interaction between Ti and Co. The Co–O distance of 2.0342(14) Å in **4** is longer than that observed for (η^2^-MesNP^i^Pr_2_)Zr(μ-O)(MesNP^i^Pr_2_)_2_Co=CPh_2_ (1.9710(16) Å) (**5**),^21^ in which the oxo ligands are only bridging between Zr and Co.

The reductive coupling of ketones (or aldehydes) to form alkenes as occurs upon addition of benzophenone to **3** is known as the McMurry reaction.^40,41^ A typical McMurry reaction involves the reduction of TiCl_4_ or TiCl_3_ with an external reductant such as Li, Na, K, Mg, Mg(Hg), Zn or LiAlH_4_, followed by the reductive coupling of ketones to alkenes by the active low valent titanium species. Stoichiometric reagents and elevated temperatures are often required, and problems of reproducibility and product selectivity (alkene *vs.* pinacol, E *vs.* Z) have limited the scope of this reaction.^40,41^ Furthermore, the mechanism of this reaction is poorly understood and operative mechanisms proceeding through carbenoid or pinacolate intermediates have been proposed.^40^

A number of ketone substrates were screened to investigate the substrate scope of the McMurry reaction promoted by complex **3**. We were pleased to find that complex **3** is reactive towards a variety of ketone substrates, affording moderate to high yields of alkene products at room temperature with relatively short reaction times, provided that stoichiometric NaI is added (Table 2). In all cases, complex **4** is formed as a byproduct of the reaction, as confirmed by ^1^H NMR spectroscopy. Asymmetric ketones gave a mixture of Z and E isomers in a *ca.* 2 : 1 ratio determined by GC-MS (entries 2–4, 7–8). It was also found that ketone substrates with more electron donating arene substituents (entries 6–9) require longer reaction times, and that only aryl-substituted ketones afforded alkene products. These observations suggest that, as with McMurry reactions in the literature, electron transfer from **3** to the ketone substrate is an essential step.

**Table 2:** Results of the reductive coupling reaction using heterobimetallic complex **3** ^*a*^

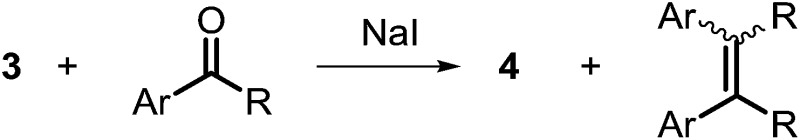			
Entry	Substrates	Time (min)	Yield (%)^*b*^
1	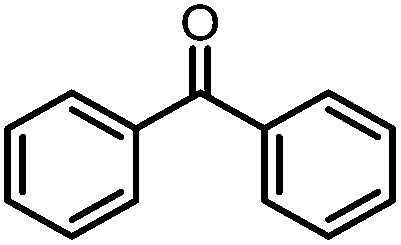	5	89
2	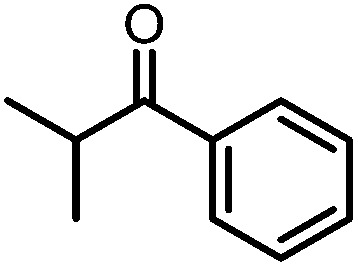	5	51
3	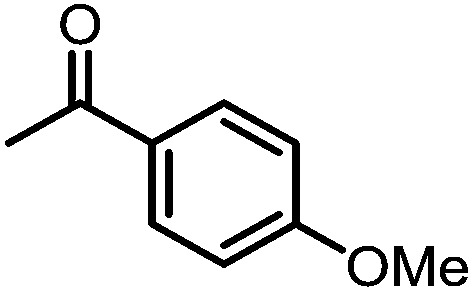	5	78
4	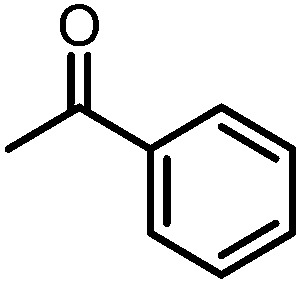	5	85
5	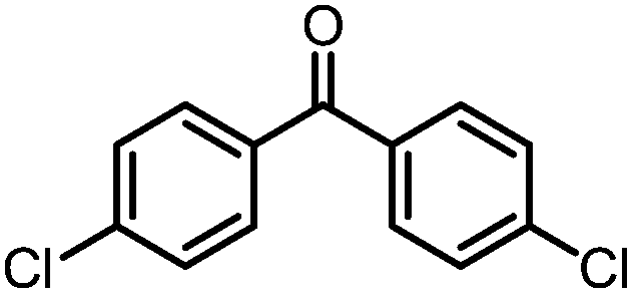	5	86
6	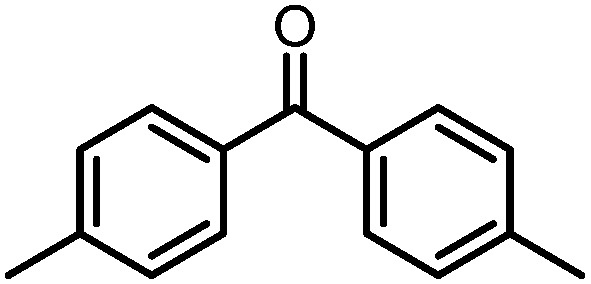	20	53
7	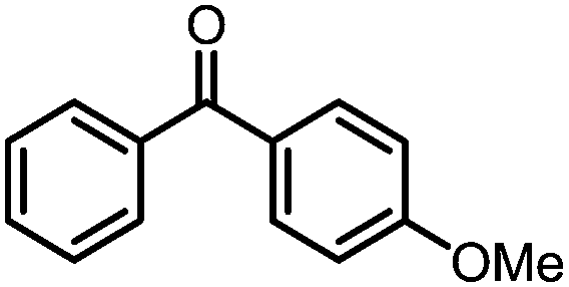	30	65
8	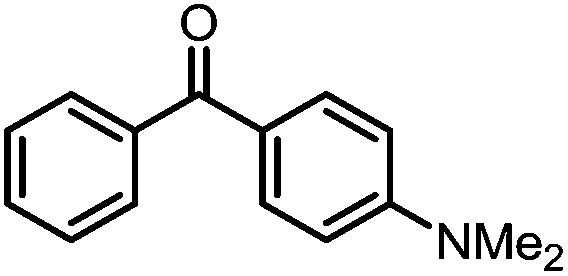	60	49
9	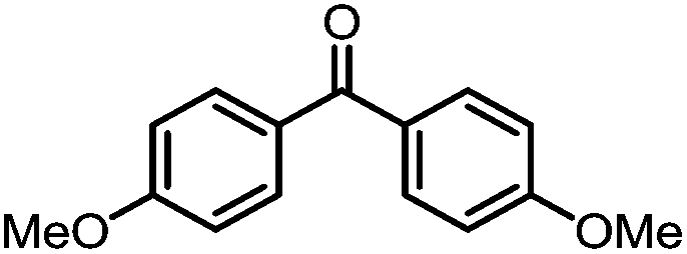	120	56

^*a*^Conditions: **3** (0.020 mmol), aryl ketone (0.020 mmol), NaI (0.040 mmol), room temperature, C_6_D_6_ (1 mL).

^*b*^Yield was determined by ^1^H NMR using hexamethylbenzene as an internal standard.

To determine the role of Ti and Co and the potential cooperative reactivity between the two metals, the reactivity of monometallic Ti and Co analogues with benzophenone was also screened. Neither **1** nor ICo(Ph_2_PNH^i^Pr)_3_ (ref. 16) shows similar reactivity with aryl ketones. A reduced monometallic titanium(iii) complex, Ti(XylNP^i^Pr_2_)_3_, was also synthesized and shows no activity to promote the reductive coupling of ketones within 3 hours. Furthermore, neither Ti(XylNP^i^Pr_2_)_3_ nor ICo(Ph_2_PNH^i^Pr)_3_ afforded the McMurry coupling product from benzophenone when Zn powder or KC_8_ were added as external reductants. These control reactions show that both Ti and Co play an essential role in the reaction.

**Scheme 4:**
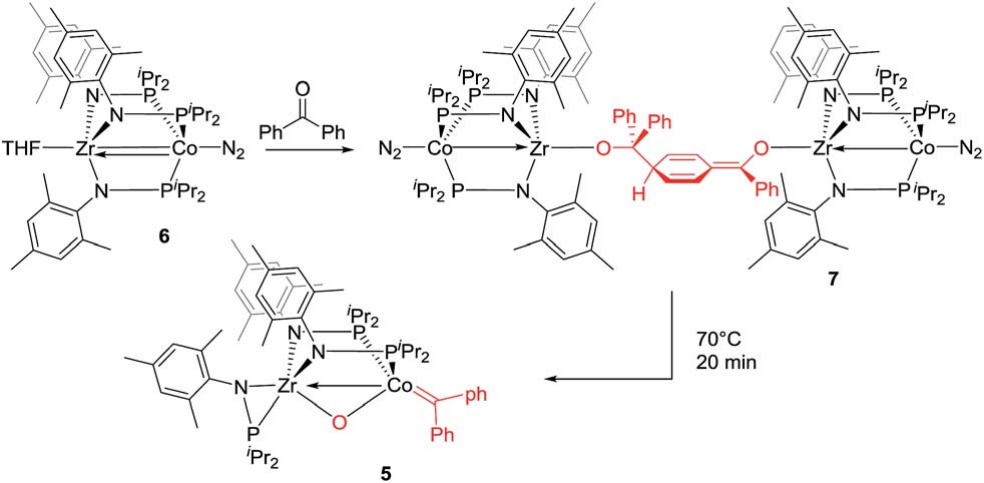
Previously reported reactivity of heterobimetallic Zr/Co complex with benzophenone.^23,24^

**Scheme 5:**
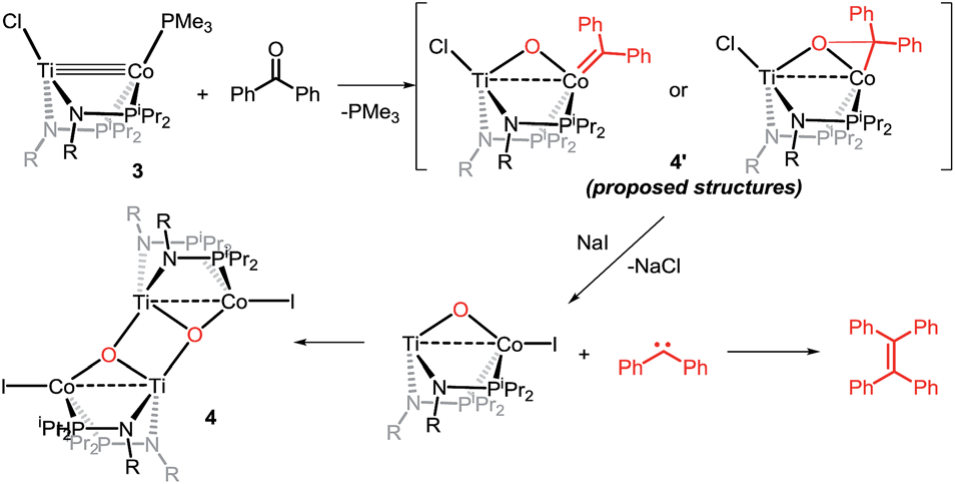
Possible reaction pathway of McMurry reaction mediated by **3**.

The mechanism by which benzophenone and other aryl ketones are reductively coupled into alkenes is intriguing. Our group previously reported that the reaction of benzophenone with heterobimetallic Zr/Co complex (THF)Zr(MesNP^i^Pr_2_)_3_CoN_2_ (**6**) leads to a benzophenone radical coupling product **7** and thermolysis of **7** affords the μ-oxo terminal carbene complex **5** (Scheme 4).^24^ Upon thermolysis at 110 °C, complex **5** decomposes, producing a mixture of tetraphenylethane, tetraphenylethylene, and diphenylmethane as the :CPh_2_ fragment is extruded. By analogy to the Zr/Co system, it is proposed that the intermediate complex **4′** obtained upon initial exposure of **3** to benzophenone is a μ-oxo/carbene complex formed upon oxidative addition of the ketone's C=O double bond (Scheme 5). Another possible structure of **4′** is a bridging μ_2_-η^1^,η^2^-ketone adduct (Scheme 5). In either case, upon addition of NaI, the :CPh_2_ fragment is released from the Co center to form the tetraphenylethylene coupled product. The resulting μ-oxo complex dimerizes to form complex **4** (Scheme 5). McMurry coupling reactions have also been shown to proceed through pinacolate intermediates; however, several key experiments have ruled out the possibility that **4′** contains a bound pinacolate. First, two separate solutions of **3** were treated with benzophenone and 4,4′-dimethylbenzophenone to form **4′** and its *p*-tolyl-substituted analogue *in situ*. Combination of these two solutions in the presence of NaI afforded a statistical distribution of alkene hetero- and homo-coupling products, namely Ph_2_C=CPh_2_, Ph_2_C=C(*p*-tolyl)_2_, and (*p*-tolyl)_2_C=C(*p*-tolyl)_2_. The crossover product could not form if a pinacolate mechanism was operative. Furthermore, addition of stoichiometric H_2_O to intermediate **4′** affords free ketone and a small amount of tetraphenylethylene, with no evidence for diol hydrolysis products that would form from a pinacolate complex.

## Conclusions

In summary, we have synthesized heterobimetallic Ti–Co complexes linked by two phosphinoamide ligands and the reduced low coordinate Ti–Co complex **3** features a strong metal–metal triple bond, with planar geometry at both Ti and Co. This low coordinate Ti–Co complex is shown to promote the reductive coupling of aryl ketones into alkenes. Future studies will focus on exploring the mechanism of this reaction and the reactivity of **3** towards other unsaturated substrates.
