# Supplementary material for: A heterobimetallic complex featuring a Ti–Co multiple bond and its application to the reductive coupling of ketones to alkenes
Source: Chem Sci. 2015 Jan 19;6(3):2044–9. doi: 10.1039/c4sc03772c (PMC5654242; doi:10.1039/c4sc03772c)
Supplement: Supplementary file 1 [file SC-006-C4SC03772C-s001.pdf]

Electronic Supplementary Material (ESI) for Chemical Science.
This journal is © The Royal Society of Chemistry 2015

# Supporting Information

# A Heterobimetallic Complex Featuring a Ti-Co Multiple Bond and Its Application to the Reductive Coupling of Ketones to Alkenes

Bing Wu, Mark W. Bezpalko, Bruce M. Foxman and Christine M. Thomas*

## Table of Contents


| | |
|---|---|
| **Experimental Section** | S2 |
| **Figure S1**. $^{1}$H NMR spectrum of **1**. | S6 |
| **Figure S2**. $^{1}$H NMR spectrum of **2**. | S6 |
| **Figure S3**. $^{1}$H NMR spectrum of **3**. | S7 |
| **Figure S4**. $^{1}$H NMR spectrum of **4**. | S7 |
| **Figure S5**. $^{1}$H NMR spectrum of **4'**. | S8 |
| **Figure S6**. $^{1}$H NMR spectrum of ($^{i}Pr_2$PNXyl)$_3$Ti. | S8 |
| **Figure S7**. Depictions and energies of the frontier molecular orbitals of **2**. | S9 |
| **Table S1**. X-ray diffraction experimental details for **2**, **3**, and **4**. | S10 |
| **Figure S8**. Fully labeled ellipsoid representation of **2**. | S11 |
| X-Ray data collection, solution, and refinement details for **2**. | S11 |
| **Figure S9**. Fully labeled ellipsoid representation of **3**. | S13 |
| X-Ray data collection, solution, and refinement details for **3**. | S13 |
| **Figure S10**. Fully labeled ellipsoid representation of **4**. | S15 |
| X-Ray data collection, solution, and refinement details for **4**. | S15 |
| XYZ Coordinates of DFT optimized geometry of **3**. | S17 |
| XYZ Coordinates of DFT optimized geometry of **2**. | S20 |
| **References** | S25 |

**General Considerations.**

All manipulations were carried out under an inert atmosphere using a nitrogen-filled glovebox or standard Schlenk techniques unless otherwise noted. All glassware was oven or flame dried immediately prior to use. Diethyl ether was obtained as HPLC grade without inhibitors; pentane, and benzene were obtained as ACS reagent grade. All protio solvents were degassed by sparging with ultra high purity argon and dried via passage through columns of drying agents using a Seca solvent purification system from Pure Process Technologies. Benzene-$d_6$ was degassed and dried over 4 Å molecular sieves before use. THF-$d_8$ was dried with $CaH_2$ and degassed before use. All NMR spectra were obtained using a Varian Inova 400 MHz instrument. $^1H$ and $^{13}C$ NMR chemical shifts were referenced to residual solvent and $^{31}P$ NMR chemical shifts were referenced to 85% $H_3PO_4$. $^iPr_2PNHXyl$[1] and $ICo(^iPrNHPPh_2)_3$[2] were synthesized using literature procedures. All other reagents and solvents were obtained from commercial sources and used without further purification. UV-vis spectra were recorded on a Cary 50 UV-vis spectrophotometer using Cary WinUV software. Elemental microanalyses were performed by Complete Analysis Laboratories, Inc., Parsippany, NJ. GC-MS data were collected on an Agilent 7890A GC System and 5975C VL MSD with a Triple-Axis Detector.

**X-ray Crystallography.**

All operations were performed on a Bruker-Nonius Kappa Apex2 diffractometer, using graphite-monochromatedMoKα radiation. All diffractometer manipulations, including data collection, integration, scaling, and absorption corrections were carried out using the Bruker Apex2 software.[3] Preliminary cell constants were obtained from three sets of 12 frames. Fully labeled diagrams and data collection and refinement details are included in Table S1 and on pages S11-S16.

**Computational Details.**

All calculations were performed using Gaussian09, Revision A.02 for the Linux operating system.[4] Density functional theory calculations were carried out using a combination of Becke's 1988 gradient-corrected exchange functional[5] and Perdew's 1986 electron correlation functional[6] (BP86). A mixed-basis set was employed, using the LANL2TZ(f) triple zeta basis set with effective core potentials for cobalt and titanium,[7] Gaussian09's internal6-311+G(d) for heteroatoms (nitrogen, phosphorus, chlorine), and Gaussian09's internal LANL2DZ basis set (equivalent to D95V[8]) for carbon and hydrogen. Using crystallographically determined geometries as a starting point, the geometries were optimized to a minimum, followed by analytical frequency calculations to confirm that no imaginary frequencies were present. Complex **2** was simplified by changing the cobalt-bound iodide ligand to chloride and performing calculations on $[(\mu\text{-}Cl)Ti(XylNP^iPr_2)_2CoCl]_2$ (**2'**). Natural atomic orbital and natural bond orbital analyses were performed using NBO Version 3.1.[9] XYZ coordinates of optimized geometries are provided on pages S17-S24.

**Synthesis of ($^iPr_2PNXyl)_2TiCl_2$ (1)**

A solution of $^iPr_2PNHXyl$ (1.42 g, 6.0 mmol) was cooled to -78 °C. To this was added $^n$BuLi (3.7 mL, 1.6 M in hexanes, 6.0 mmol) dropwise over 10 min. The resulting yellow solution was warmed to room temperature and stirred for 2 h. The solution was then cooled again to -78 °C, and $TiCl_4$ (330 µL, 3.0 mmol) was added. The reaction mixture was warmed to room temperature and stirred for 12 h to ensure completion of reaction. LiCl was removed by filtration and the volatiles were removed from the solution in vacuo. The crude dark material was extracted with pentane and slow evaporation of a concentrated pentane solution of **1** at room temperature yielded yellow crystals of **1** (1.13 g, 64%). $^1H$ NMR (δ in ppm, $C_6D_6$, 293 K, 400 MHz): 6.61 (s, 4H, *o*-Ar), 6.42 (s, 2H, *p*-Ar), 2.44 (m, 4H, $C\mathit{H}(CH_3)_2$), 2.07 (s, 12H, Ar-$CH_3$), 1.28 (m, 12H, $CH(C\mathit{H}_3)_2$), 1.12 (m, 12H, $CH(C\mathit{H}_3)_2$); $^{31}P\{^1H\}$ NMR (δ in ppm, $C_6D_6$, 293 K, 162 MHz): 6.8 (s); $^{13}C\{^1H\}$ NMR (δ in ppm, $C_6D_6$, 293 K, 100.63 MHz): 153.4, 138.1, 126.3, 121.4, 29.6, 21.8, 20.6, 20.0. UV-vis ($Et_2O$) $\lambda_{max}$ (nm) (ε [$mol^{-1}cm^{-1}$]): 250 (2590), 300 (920), 607 (105). Anal. Calcd for $C_{28}H_{46}N_2P_2TiCl_2$: C, 56.87; H, 7.84; N, 4.74. Found: C, 56.96; H, 7.91; N, 4.63.

**Synthesis of [(*µ*-Cl)Ti($XylNP^iPr_2)_2CoI]_2$ (2)**

Solid **1** (0.590g, 1.00 mmol), solid $CoI_2$ (0.313 g, 1.00 mmol), and Zn powder (0.131 g, 2.00 mmol) were combined in THF (15 mL) and stirred for 24 h at room temperature. The resulting brown solution was filtered through Celite and solvent was removed from the filtrate in vacuo. The crude dark material was extracted with $C_6H_6$ and slow evaporation of a concentrated $Et_2O$ solution of **2** at room temperature yielded brown crystals of **2** (0.460 g, 62.1%). Single crystals of **2** suitable for X-ray diffraction were grown from a concentrated $Et_2O$ solution at room temperature. $^1H$ NMR (400 MHz, $C_6D_6$): δ 6.62, 3.70, 2.20, 0.55, -2.06 (methine protons were not observed due to broadening). Evans' method ($C_6D_6$): 3.11 µB. UV-vis ($Et_2O$) $\lambda_{max}$ (nm) (ε [$mol^{-1}cm^{-1}$]): 250 (33000), 310 (6000), 590 (645), 750 (180). Anal. Calcd for $C_{56}H_{92}N_4P_4Ti_2Co_2I_2Cl_2$: C, 45.34; H, 6.25; N, 3.78. Found: C, 45.40; H, 6.58; N, 3.75.

**Synthesis of ClTi($XylNP^iPr_2)_2CoPMe_3$ (3)**

A solution of **2** (0.148 g, 0.10 mmol) and neat $PMe_3$ (30 µL, 0.30 mmol) in THF (10 mL) was added dropwise to solid $KC_8$ (0.040 g, 0.30 mmol) over 5 min. The resulting mixture was continuously stirred for 3 h to ensure completion of the reaction. The insoluble graphite byproduct was removed via filtration and volatiles were removed from the filtrate in vacuo. The crude brown materials were extracted into $Et_2O$. Slow evaporation of the concentrated $Et_2O$ solution of **3** at room temperature yielded analytically pure red blocks of **3** (0.089 g, 65%). Single crystals of **3** suitable for X-ray diffraction were grown from a concentrated $Et_2O$ solution at room temperature. $^1H$ NMR (400 MHz, $C_6D_6$): δ 6.45 (s, 2H, *p*-Ar), 6.43 (s, 4H, *o*-Ar), 3.26 (m, 4H, $C\mathit{H}(CH_3)_2$), 2.10 (s, 12H, Ar-$CH_3$), 1.71 (m, 9H, $PMe_3$), 1.32 (m, 12H, $CH(C\mathit{H}_3)_2$), 1.21(m, 12H, $CH(C\mathit{H}_3)_2$); $^{31}P\{^1H\}$ NMR ($C_6D_6$, 293 K, 162 MHz): δ 44.6(d), -21.0(t); $^{13}C\{^1H\}$ NMR ($C_6D_6$, 293 K, 100.63 MHz): δ 149.7, 139.2, 123.3, 119.5, 32.33,

28.90, 22.1, 20.29. UV-vis ($Et_2O$) $\lambda_{max}$ (nm) ($\varepsilon$ [$mol^{-1}cm^{-1}$]): 250 (9300), 300 (2690), 583 (195). Anal. Calcd for $C_{31}H_{55}N_2P_3TiCoCl$: C, 53.89; H, 8.02; N, 4.05. Found: C, 53.67; H, 8.14; N, 4.07.

**Synthesis of [Ti($\mu_3$-O)(XylNP$^i$Pr$_2$)$_2$CoI]$_2$ (4)**

A solution of **3** (69 mg, 0.10 mmol) in diethyl ether was added to solid 4,4'-dichlorobenzophone (25 mg, 0.10 mmol) and NaI (30 mg, 0.20 mmol). The reaction mixture was stirred for 5 min, resulting in the formation of a purple precipitate. The volatiles were removed in vacuo and the remaining solids were extracted into benzene. Slow evaporation of the concentrated $C_6H_6$ solution of **4** at room temperature afforded purple crystals of **4** (42 mg, 57%).$^1$H NMR (400 MHz, THF-$d_8$): $\delta$ 7.92, 5.43, 3.95, 3.34, -1.81, -4.89, -7.46. UV-vis ($Et_2O$) $\lambda_{max}$ (nm) ($\varepsilon$ [$mol^{-1}cm^{-1}$]): 250 (10300), 300(2930), 370 (1520), 420 (950). Evans' method ($C_6D_6$): 5.32 μB. Anal. Calcd for $C_{56}H_{92}N_2P_2Ti_2Co_2O_2I_2$: C, 46.56; H, 6.42; N, 3.88. Found: C, 46.38; H, 6.37; N, 3.58.

**Synthesis of ($^i$Pr$_2$PNXyl)$_3$Ti**

A solution of $^i$Pr$_2$PNHXyl (1.42 g, 6.0 mmol) was cooled to -78 °C. To this was added $^n$BuLi (5.0 mL, 1.6 M in hexanes, 8.0 mmol) dropwise over 10 min. The resulting yellow solution was warmed to room temperature and stirred for 2 h. The solution was then cooled again to -78 °C, and $TiCl_4$ (220 μL, 2.0 mmol) was added. The reaction mixture was warmed to room temperature and stirred for 12 h to ensure completion of reaction. LiCl was removed by filtration and the volatiles were removed from the solution in vacuo. The crude dark material was extracted with pentane and slow evaporation of a concentrated pentane solution of **8** at room temperature yielded light green crystals of **8** (0.832 g, 55%). $^1$H NMR ($\delta$ in ppm, $C_6D_6$, 293 K, 400 MHz): 7.94, 3.13, 2.68, 1.66, 0.30, -2.66. UV-vis ($Et_2O$) $\lambda_{max}$ (nm) ($\varepsilon$ [$mol^{-1}cm^{-1}$]): 250 (2630), 305 (520), 530 (130). Evans' method ($C_6D_6$): 1.82 $\mu_B$. Anal. Calcd for $C_{42}H_{69}N_3P_3Ti$: C, 66.66; H, 9.19; N, 5.55. Found: C, 66.62; H, 9.26; N, 5.50.

**Yield Determination of McMurry Reactions using $^1$H NMR Spectroscopy**.

Aryl ketones (0.020 mmol) and NaI (0.040 mmol, 6.0 mg) were added to a stirring $Et_2O$ (2 mL) solution of **3** (0.020 mmol, 14.0 mg) at room temperature. The remaining NaI was removed via filtration after the completion of the reactions and hexamethylbenzene (0.010 mmol, 16.2 mg) was added to the solution. $^1$H NMR yields were determined via integration against hexamethylbenzene.

**Reaction of 4' with $H_2O$**

A solution of **3** (69 mg, 0.10 mmol) in diethyl ether was added to solid benzophone (18 mg, 0.10 mmol) and charged to a Schlenk tube containing a stir bar and sealed with a Teflon valve. A 0.10 M solution of $H_2O$ in THF (1 mL, 0.10 mmol), prepared from 90 μL of $N_2$-sparged deionized water and 50 mL of THF, was added dropwise via a syringe. The reaction mixture was transferred to a scintillation vial, and volatiles were removed in vacuo. The remaining solid was extracted into $Et_2O$ for GC/MS

analysis.

**Reaction of 3 with benzophenone and 4,4'-dimethylbenzophenone**

Two solutions of **3** (69 mg, 0.10 mmol) in diethyl ether were added to two separate vials containing solid benzophenone (18 mg, 0.10 mmol) and solid 4,4'-dimethylbenzophenone (21 mg, 0.10 mmol), respectively. Both solutions were stirred at room temperature for 5 min and then combined together. To the resulting reaction mixture, NaI (60 mg, 0.40 mmol) was added. The mixture was stirred at room temperature for another 5 min and volatiles were removed in vacuo. The remaining solid was extracted into $Et_2O$ for GC/MS analysis.

**Figure S1.** $^1H$ NMR spectrum of **1**

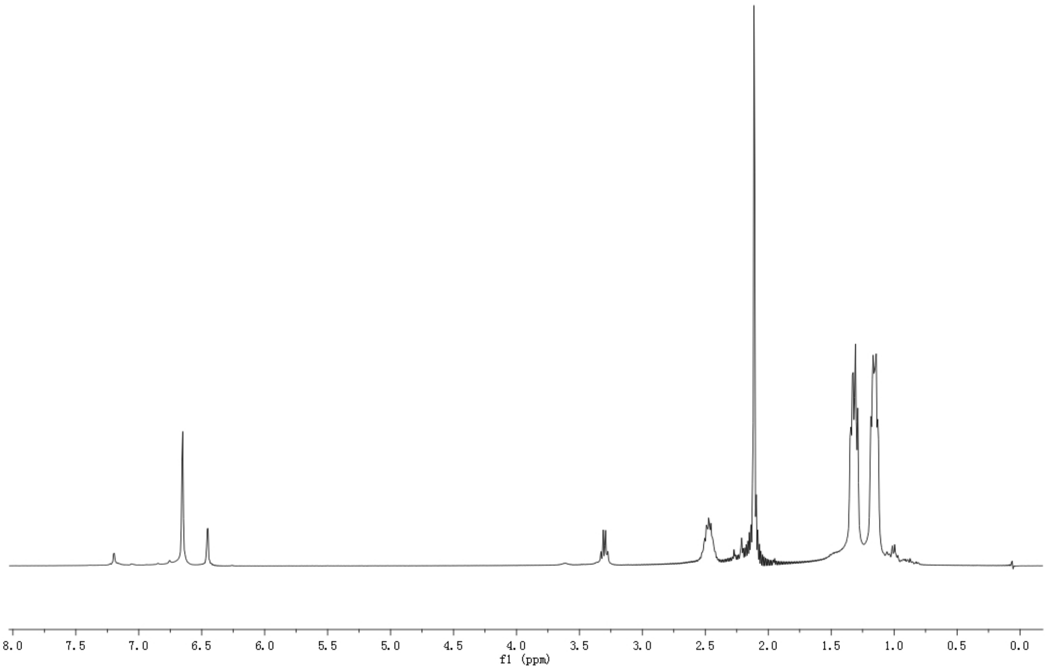


**Figure S2.** $^{1}$H NMR spectrum of **2**

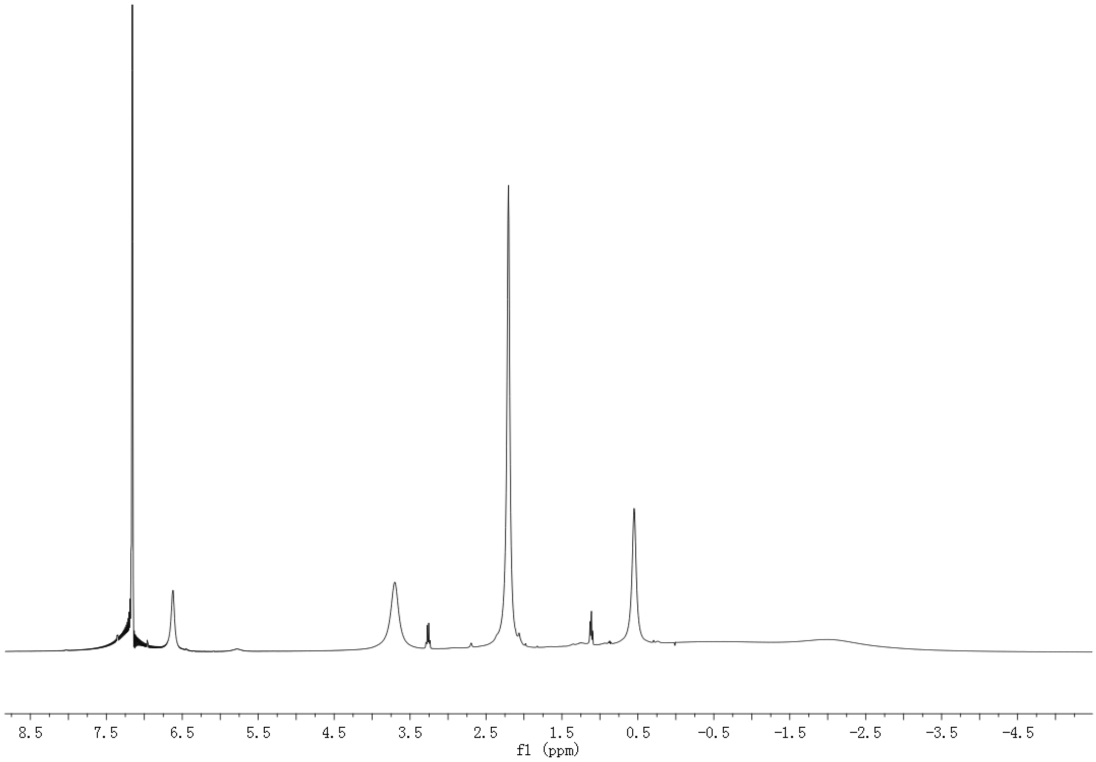


**Figure S3.** $^{1}$H NMR spectrum of **3**

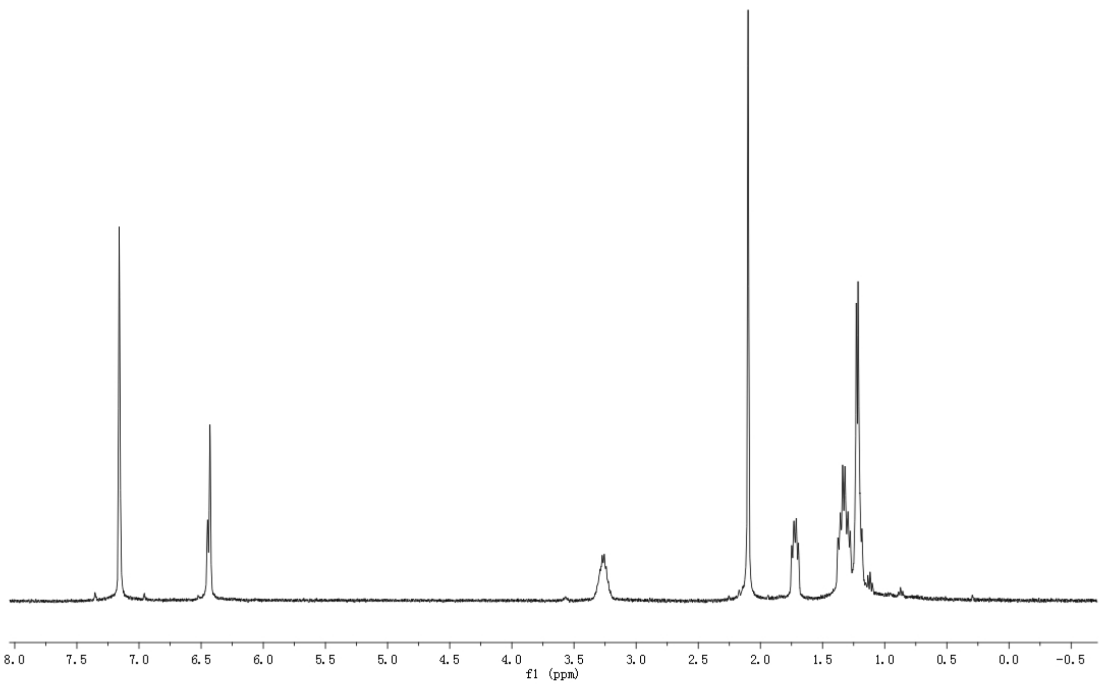


**Figure S4.** $^{1}$H NMR spectrum of **4**

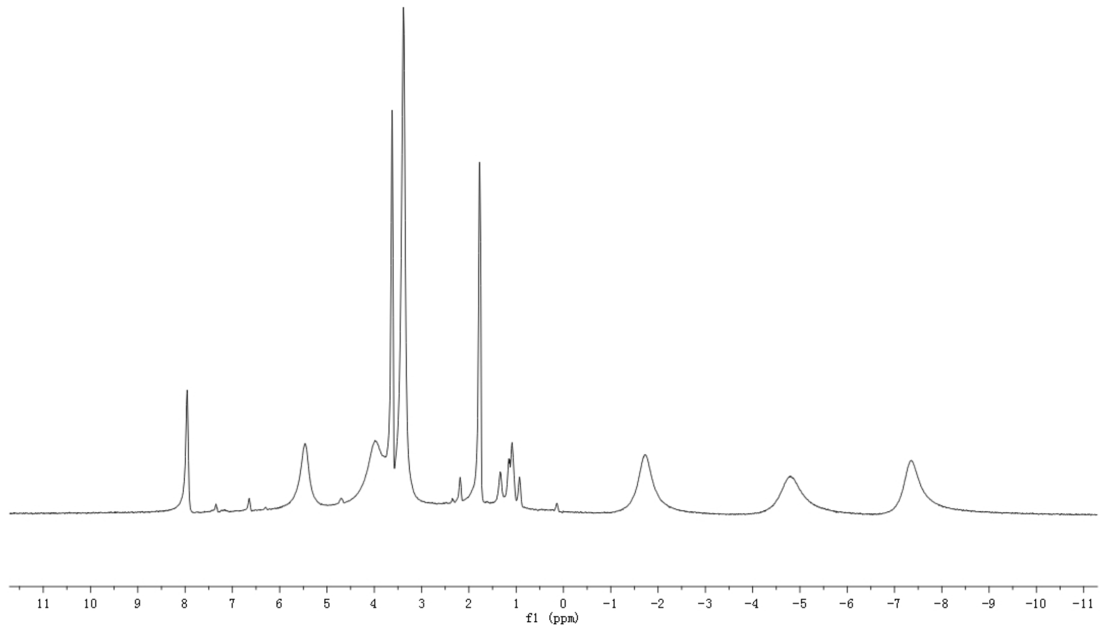


**Figure S5**. $^{1}$H NMR spectrum of **4’**.

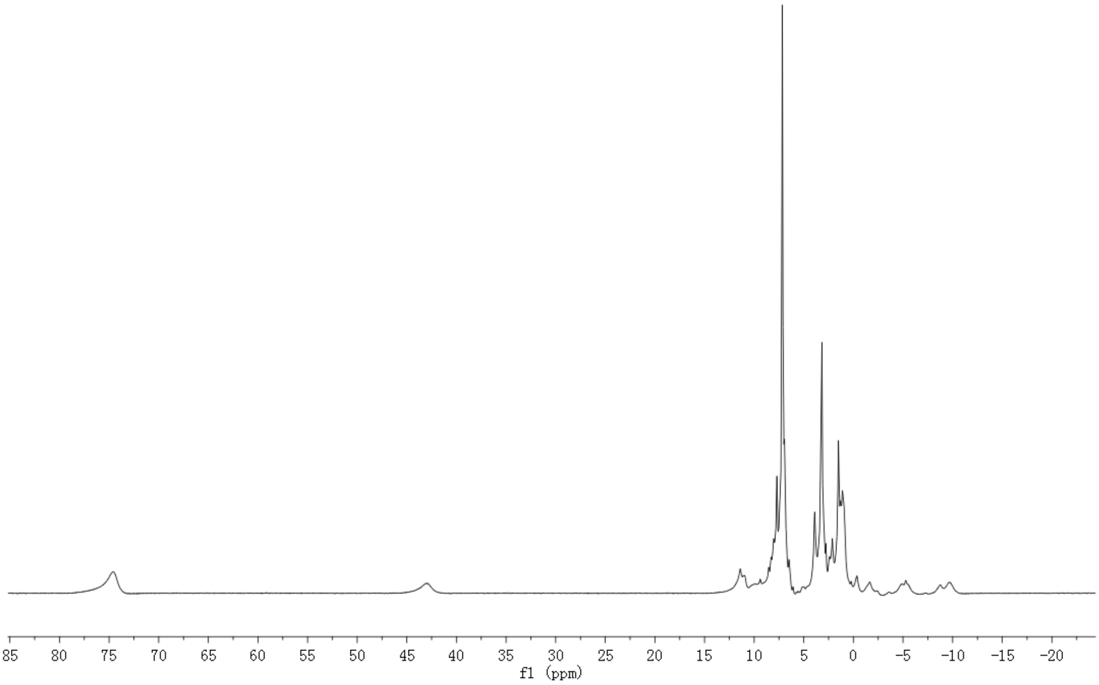


**Figure S6**. $^{1}$H NMR spectrum of **8**.

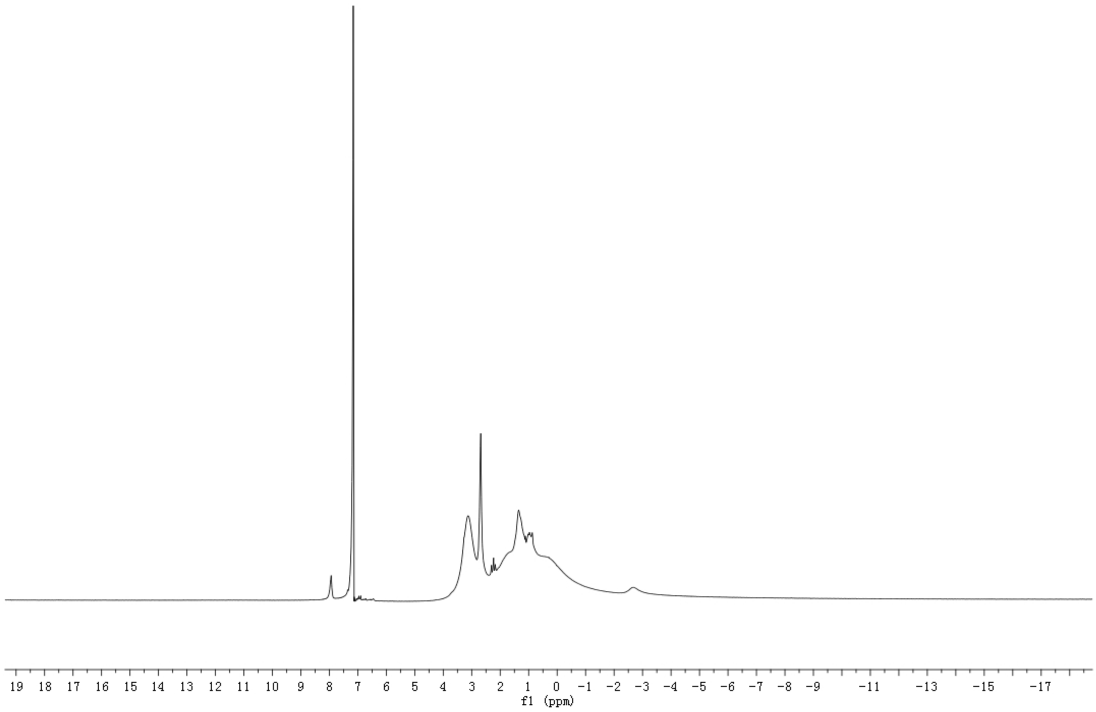


**Figure S7**. Depictions and energies of the frontier molecular orbitals of **2**.

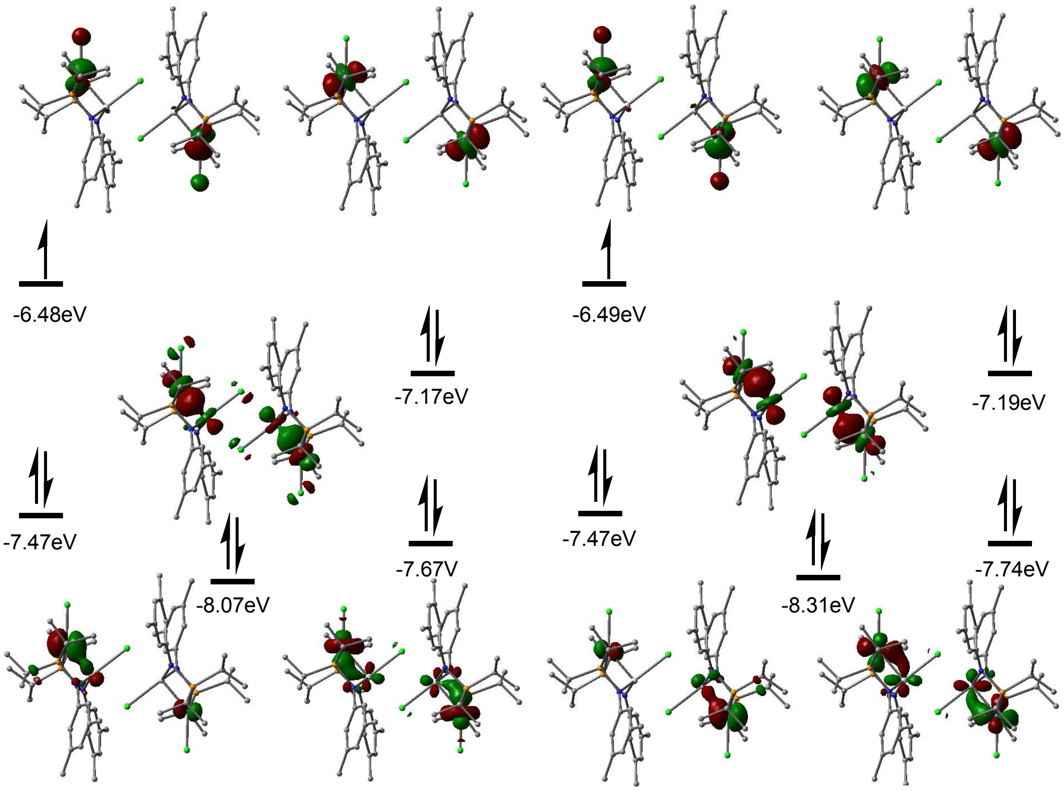


**Table S1.** X-ray Diffraction Experimental Details for complexes **2**, **3** and **4**.[a]

| | **2** | **3** | **4** |
|---|---|---|---|
| chemical formula | $C_{56}H_{92}Cl_2Co_2I_2N_4P_4Ti_2$ | $C_{31}H_{55}ClCoN_2P_3Ti$ | $C_{68}H_{104}Co_2I_2N_4O_2PTi_2$ |
| fw | 1483.64 | 691.00 | 1600.96 |
| T(K) | 120 | 120 | 120 |
| λ(Å) | 0.71073 | 0.71073 | 0.71073 |
| a(Å) | 18.6991(13) | 9.0138(7) | 12.5912(5) |
| b(Å) | 16.4217(10) | 18.8395(13) | 13.4571(5) |
| c(Å) | 21.8753(15) | 21.5085(16) | 13.5956(5) |
| α(°) | 90 | 90 | 110.6819(17) |
| β(°) | 90 | 90.540(4) | 94.623(2) |
| γ(°) | 90 | 90 | 117.1503(16) |
| V(Å$^3$) | 6717.3(8) | 3652.3(3) | 1835.07(7) |
| space group | *Pbca* | $P2_1/n$ | $P\bar{1}$ |
| Z / Z' | 4 / 0.5 | 4/ 1 | 1/ 0.5 |
| $D_{calc}$ (g/cm$^3$) | 1.47 | 1.26 | 1.45 |
| μ (cm$^{-1}$) | 18.45 | 8.99 | 16.26 |
| $R_1$ (I > 2σ) | 0.0246 | 0.0584 | 0.0327 |
| $wR_2$ (all data) | 0.0573 | 0.1820 | 0.0890 |

[a] $R1 = \Sigma ||F_o| - |F_o|| / \Sigma |F_o|$; $wR2 = \{\Sigma [w(F_o^2 - F_c^2)^2] / \Sigma [w(F_o^2)^2]\}^{1/2}$

**Figure S8.** Fully labeled ellipsoid representation of $[(\mu\text{-Cl})Ti(XylNP^iPr_2)_2CoI]_2$ (**2**)

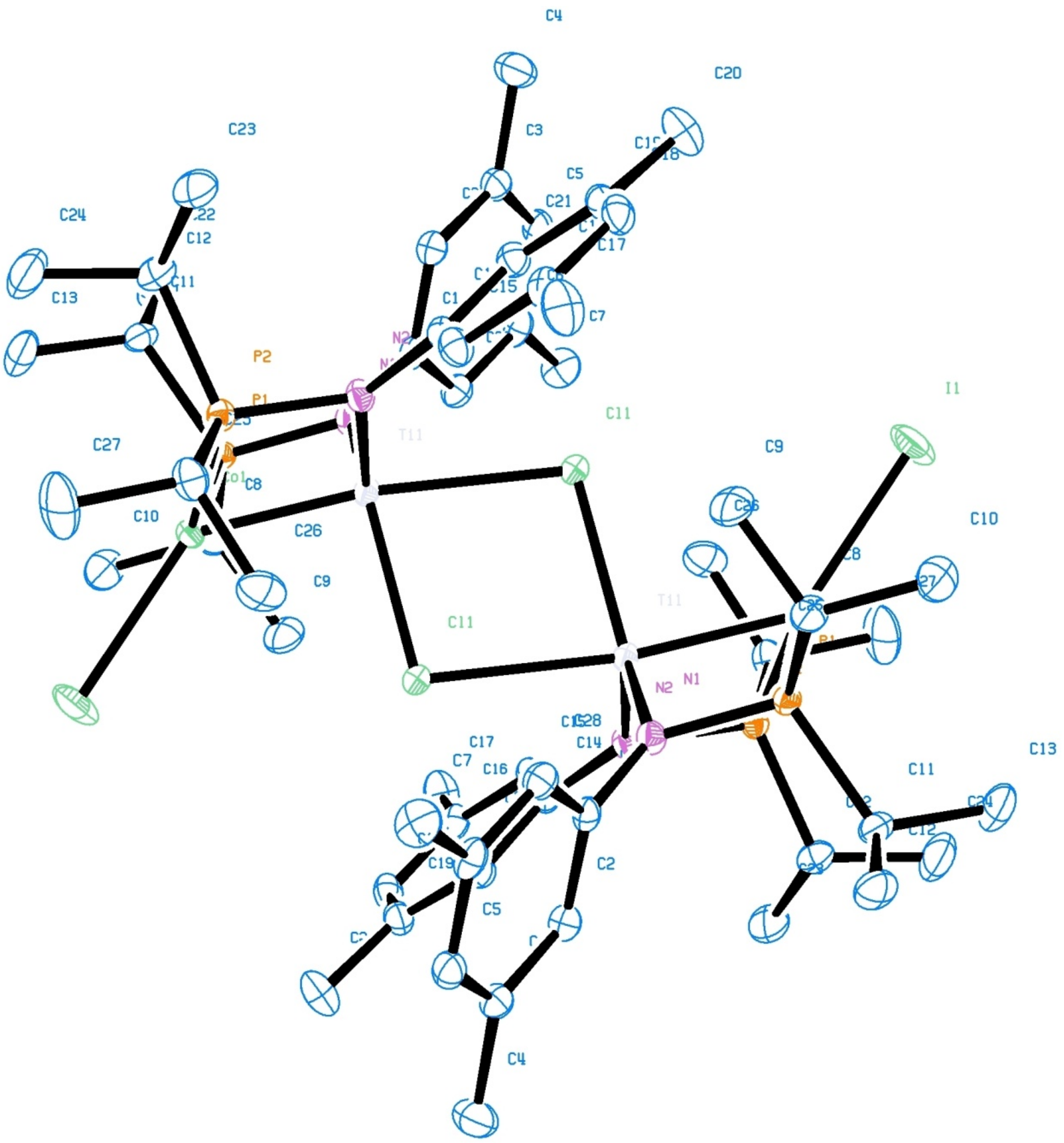


X-Ray data collection, solution, and refinement for **2**

All operations were performed on a Bruker-Nonius Kappa Apex2 diffractometer, using graphite-monochromated MoKα radiation. All diffractometer manipulations, including data collection, integration, scaling, and absorption corrections were carried out using the Bruker Apex2 software.[3] Preliminary cell constants were obtained from three sets of 12 frames.Data collection was carried out at 120K, using a frame time of 10 sec and a detector distance of 60 mm. The optimized strategy used for data collection consisted of two phi and two omega scan sets, with 0.5° steps in phi or omega; completeness was 99.9%. A total of 1124 frames were collected. Final cell constants were obtained from the xyz centroids of 9921 reflections after integration.

From the systematic absences, the observed metric constants and intensity statistics, space group *Pbca* was chosen initially; subsequent solution and refinement confirmed the correctness of this choice. The structure was solved using *SuperFlip*,[10] and refined (full-matrix-least squares) using the Oxford University *Crystals for Windows*

program.[11] The asymmetric unit contains one-half of molecule of the complex dimer ($Z$ = 4; $Z'$=½). All non-hydrogen atoms were refined using anisotropic displacement parameters. After location of H atoms on electron-density difference maps, the H atoms were initially refined with soft restraints on the bond lengths and angles to regularize their geometry (C---H in the range 0.93--0.98 Å and $U_{iso}$(H) in the range 1.2-1.5 times $U_{eq}$ of the parent atom), after which the positions were refined with riding constraints.[12] The final least-squares refinement converged to $R_1$ = 0.0246 ($I$> 2σ($I$), 7718 data) and $wR_2$ = 0.0573 ($F^2$, 9805 data, 325 parameters). The final CIF is available as supporting material.

**Figure S9.** Fully labeled ellipsoid representation of $ClTi(XylNP^iPr_2)_2CoPMe_3$ (**3**)

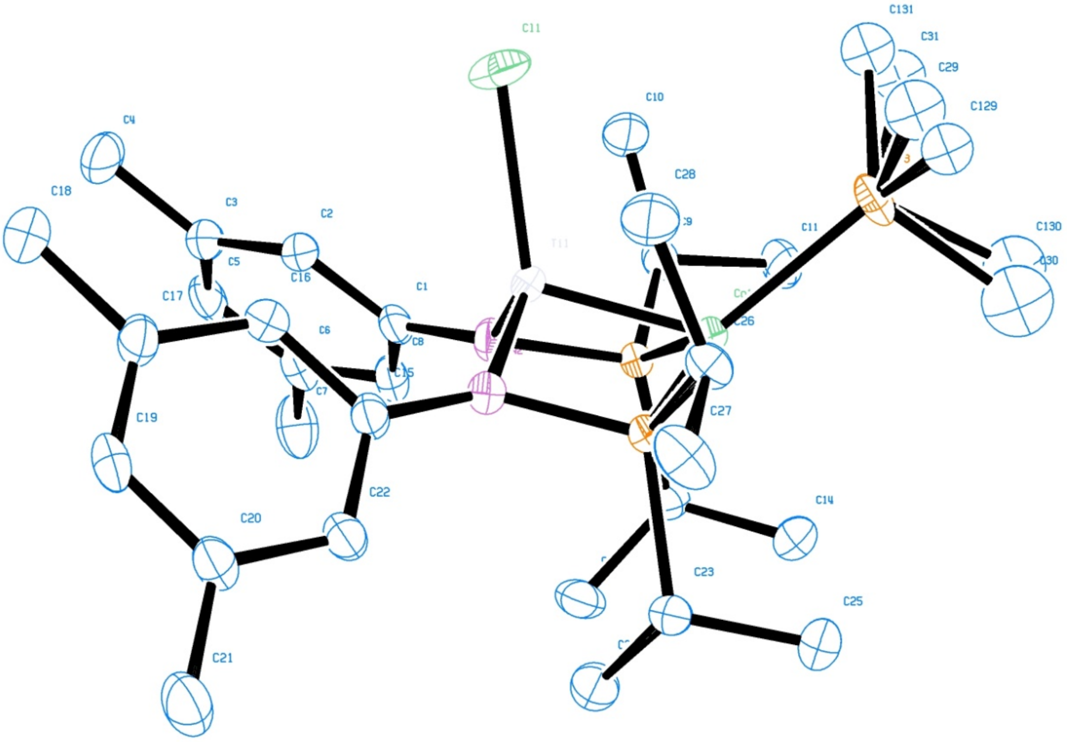


X-Ray data collection, solution, and refinement for **3**

All operations were performed on a Bruker-Nonius Kappa Apex2 diffractometer, using graphite-monochromated MoK$\alpha$ radiation. All diffractometer manipulations, including data collection, integration, scaling, and absorption corrections were carried out using the Bruker Apex2 software.[3] Preliminary cell constants were obtained from three sets of 12 frames. Data collection was carried out at 120K, using a frame time of 20 sec and a detector distance of 60 mm. The optimized strategy used for data collection consisted of three phi and five omega scan sets, with 0.5° steps in phi or omega; completeness was 96.3%. A total of 2419 frames were collected. Final cell constants were obtained from the xyz centroids of 9940 reflections after integration.

From the systematic absences, the observed metric constants and intensity statistics, space group $P2_1/n$ was chosen initially; subsequent solution and refinement confirmed the correctness of this choice. The structure was solved using *SIR-92*,[13] and refined (full-matrix-least-squares) using the Oxford University *Crystals for Windows* program.[11] The asymmetric unit contains one molecule of the complex ($Z$ = 4; $Z'$=1). All ordered non-hydrogen atoms were refined using anisotropic displacement parameters. After location of H atoms on electron-density difference maps, the H atoms were initially refined with soft restraints on the bond lengths and angles to regularize their geometry (C---H in the range 0.93--0.98 Å and $U_{iso}$(H) in the range 1.2-1.5 times $U_{eq}$ of the parent atom), after which the positions were refined with

riding constraints.[12] A plot of $F_{obs}$ vs $F_{calc}$ revealed an unusual number of reflections for which $F_{obs}$ >>$F_{calc}$, a likely indication of twinning. A Rotax analysis[14] revealed TLQS twinning,[15] with an obliquity of 0.54°, twin law (1 0 0 / 0 -1 0 / -0.045 0 -1), and an overlap tolerance parameter of 0.025 $Å^{-1}$. The sum of the twin components ratio was constrained to sum to 1.0, with a final value of the major component of 0.7168(15). The methyl groups on the trimethylphosphine were found to be disordered, and the disorder was modeled as a two-component disorder. Occupancies of major and minor component atoms, [C(29,30,31)/C(129,130,131)], respectively, were constrained to sum to 1.0; the major component occupancy refined to a value of 0.50(3). All the modeled disordered components were refined using isotropic displacement parameters. The final least-squares refinement converged to $R_1$ = 0.0584 ($I$> $2\sigma(I)$, 7540 data) and $wR_2$ = 0.1820 ($F^2$, 10380 data, 351 parameters). The final CIF is available as supporting material.

**Figure S10.** Fully labeled ellipsoid representation ofTi($\mu_3$-O)(XylNP$^i$Pr$_2$)$_2$CoI]$_2$ (**4**)

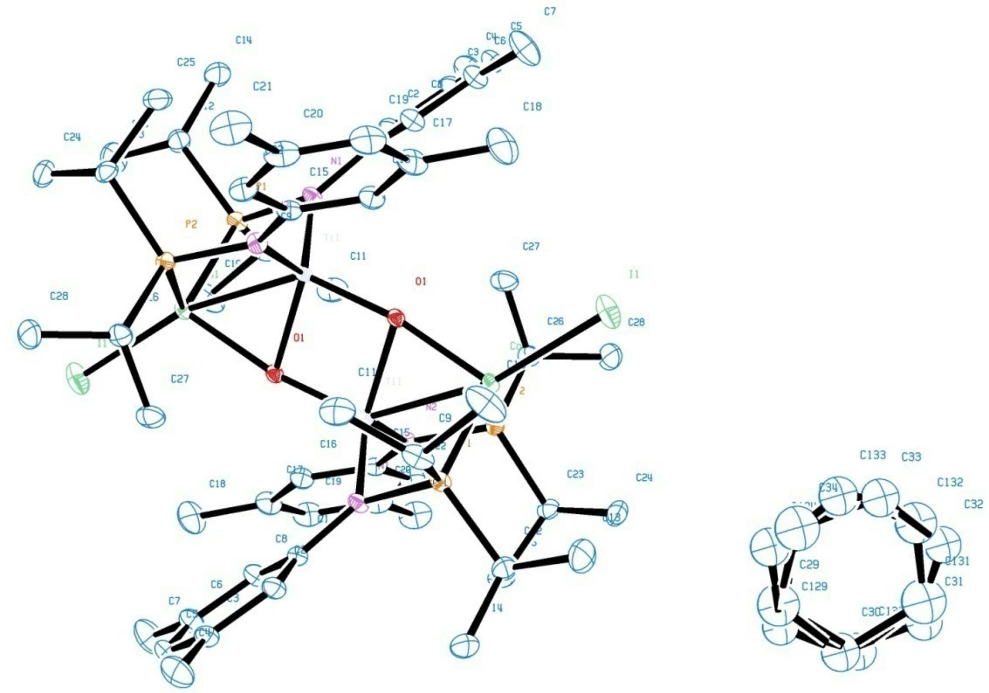


X-Ray data collection, solution, and refinement for **4**

All operations were performed on a Bruker-Nonius Kappa Apex2 diffractometer, using graphite-monochromated MoK$\alpha$ radiation. All diffractometer manipulations, including data collection, integration, scaling, and absorption corrections were carried out using the Bruker Apex2 software.[3] Preliminary cell constants were obtained from three sets of 12 frames. Data collection was carried out at 120K, using a frame time of 10 sec and a detector distance of 60 mm. The optimized strategy used for data collection consisted of six phi and three omega scan sets, with 0.5° steps in phi or omega; completeness was 99.6 %.A total of 3639 frames were collected. Final cell constants were obtained from the xyz centroids of 9901 reflections after integration.

From the systematic absences, the observed metric constants and intensity statistics, space group $P\bar{1}$ was chosen initially; subsequent solution and refinement confirmed the correctness of this choice. The structure was solved using *SuperFlip*,[10] and refined (full-matrix-least squares) using the Oxford University *Crystals for Windows* program.[11] The asymmetric unit contains one half-molecule of the complex dimer and a benzene molecule (for the dimer, $Z$ = 1; $Z'$ = 0.5). All ordered non-hydrogen atoms were refined using anisotropic displacement parameters. After location of H atoms on electron-density difference maps, the H atoms were initially refined with soft restraints on the bond lengths and angles to regularize their geometry (C---H in the range 0.93--0.98 Å and $U_{iso}$(H) in the range 1.2-1.5 times $U_{eq}$ of the parent atom), after which the positions were refined with riding constraints.[12] The benzene solvent was found to be disordered and was modeled as a two-component disorder. Occupancies of major and minor component C atoms, C(29,30,31,32,33,34) and

C(129,130,131,132,133,134), respectively, were constrained to sum to 1.0; the major component occupancy refined to a value of 0.573(13). The modeled disordered components were refined using isotropic displacement parameters. Distance and angle restraints were applied to the modeled disordered benzene solvent; details appear in the CIF file. The final least-squares refinement converged to $R_1 = 0.0327$ ($I > 2\sigma(I)$, 8716 data) and $wR_2 = 0.0890$ ($F^2$, 10702 data, 374 parameters). The final CIF is available as supporting material.

XYZ Coordinates of DFT optimized geometry of **3**

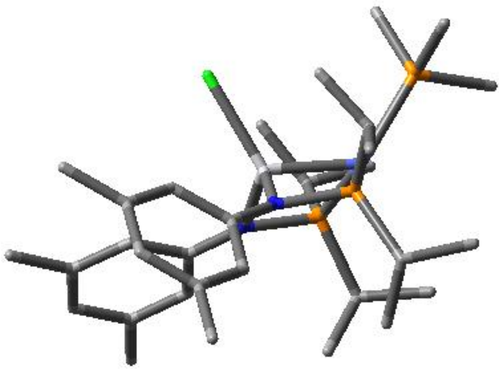

| Symbol | X | Y | Z |
|---|---|---|---|
| Co | -0.418394 | -1.813998 | 0.041023 |
| Ti | -0.014720 | 0.093915 | -0.720940 |
| P | -2.214549 | -0.558404 | 0.592579 |
| N | -1.589589 | 0.965679 | 0.110852 |
| C | -2.152729 | 2.261099 | 0.076793 |
| C | -3.358590 | 2.614281 | 0.751369 |
| C | -3.870149 | 3.934158 | 0.701446 |
| C | -5.178895 | 4.276615 | 1.403780 |
| C | -3.157527 | 4.924297 | -0.021781 |
| C | -1.953578 | 4.604006 | -0.704302 |
| C | -1.465631 | 3.279389 | -0.647062 |
| C | -1.206532 | 5.663112 | -1.505425 |
| C | -2.704676 | -0.551708 | 2.430263 |
| C | -1.920745 | 0.524404 | 3.222464 |
| C | -2.475719 | -1.971153 | 3.019055 |
| C | -3.907918 | -0.663271 | -0.260243 |
| C | -3.770562 | -0.339502 | -1.767203 |
| C | -4.599389 | -2.030687 | -0.023649 |
| P | 1.734559 | -1.403426 | 0.600804 |
| N | 1.772930 | 0.241343 | 0.125412 |
| C | 2.801109 | 1.219525 | 0.081725 |
| C | 3.683510 | 1.461082 | 1.169829 |
| C | 4.666427 | 2.482416 | 1.103409 |
| C | 5.602366 | 2.719207 | 2.283092 |
| C | 4.757104 | 3.276217 | -0.066310 |
| C | 3.884115 | 3.062259 | -1.168965 |
| C | 2.917504 | 2.035118 | -1.084554 |
| C | 4.000884 | 3.918004 | -2.424196 |
| C | 2.343725 | -1.650677 | 2.376301 |
| C | 2.257292 | -3.160103 | 2.736591 |
| C | 1.580515 | -0.789675 | 3.406752 |
| C | 3.124458 | -2.247996 | -0.399185 |

| | | | |
|---|---|---|---|
| C | 4.573760 | -2.023315 | 0.112847 |
| C | 3.016940 | -1.816718 | -1.885064 |
| Cl | -0.029645 | 0.272983 | -3.023885 |
| P | -0.731700 | -3.664843 | -1.174161 |
| C | -1.703020 | -3.524769 | -2.753590 |
| C | 0.713882 | -4.677000 | -1.787001 |
| C | -1.654197 | -5.023844 | -0.278566 |
| H | -3.905386 | 1.859542 | 1.329063 |
| H | -5.209900 | 5.340097 | 1.702638 |
| H | -6.046986 | 4.095550 | 0.738547 |
| H | -5.324781 | 3.661927 | 2.311189 |
| H | -3.541658 | 5.952611 | -0.055400 |
| H | -0.537860 | 3.024264 | -1.177823 |
| H | -0.113685 | 5.582875 | -1.357882 |
| H | -1.401201 | 5.549143 | -2.590355 |
| H | -1.518773 | 6.682429 | -1.215499 |
| H | -3.789983 | -0.315048 | 2.476767 |
| H | -2.103325 | 0.401243 | 4.308888 |
| H | -2.220589 | 1.545730 | 2.933609 |
| H | -0.837402 | 0.434649 | 3.040275 |
| H | -2.741524 | -1.990131 | 4.094567 |
| H | -3.077602 | -2.743255 | 2.504295 |
| H | -1.410061 | -2.255976 | 2.922210 |
| H | -4.535676 | 0.123745 | 0.206578 |
| H | -4.751290 | -0.451284 | -2.270959 |
| H | -3.414955 | 0.692272 | -1.928125 |
| H | -3.048989 | -1.015504 | -2.260129 |
| H | -5.584735 | -2.044054 | -0.529329 |
| H | -4.773743 | -2.237704 | 1.049125 |
| H | -3.998377 | -2.859430 | -0.442328 |
| H | 3.592070 | 0.863967 | 2.084496 |
| H | 6.254732 | 3.593180 | 2.109424 |
| H | 5.034939 | 2.900206 | 3.216185 |
| H | 6.255069 | 1.842316 | 2.462090 |
| H | 5.514469 | 4.070037 | -0.122896 |
| H | 2.253758 | 1.834485 | -1.936770 |
| H | 3.254304 | 3.623158 | -3.182172 |
| H | 3.848090 | 4.990266 | -2.193832 |
| H | 5.004786 | 3.823523 | -2.881907 |
| H | 3.409028 | -1.346010 | 2.382255 |
| H | 2.603555 | -3.321527 | 3.776167 |
| H | 1.212031 | -3.520174 | 2.671233 |
| H | 2.879161 | -3.789911 | 2.073572 |
| H | 2.100189 | -0.822131 | 4.385110 |

| | | | |
|---|---|---|---|
| H | 0.556722 | -1.174225 | 3.556871 |
| H | 1.506050 | 0.265752 | 3.088818 |
| H | 2.891536 | -3.331986 | -0.332771 |
| H | 5.286688 | -2.511167 | -0.581758 |
| H | 4.822677 | -0.947765 | 0.146889 |
| H | 4.747932 | -2.455400 | 1.115774 |
| H | 3.669590 | -2.455742 | -2.512301 |
| H | 3.342393 | -0.768622 | -2.010033 |
| H | 1.984071 | -1.886098 | -2.272031 |
| H | -2.743281 | -3.231610 | -2.528956 |
| H | -1.254180 | -2.735763 | -3.383546 |
| H | -1.709205 | -4.481242 | -3.311729 |
| H | 1.340374 | -4.979754 | -0.928040 |
| H | 1.334970 | -4.077476 | -2.475408 |
| H | 0.370236 | -5.587133 | -2.317430 |
| H | -1.076344 | -5.342864 | 0.608879 |
| H | -2.637587 | -4.657509 | 0.064457 |
| H | -1.806340 | -5.903052 | -0.935387 |

XYZ Coordinates of DFT optimized geometry of **2**

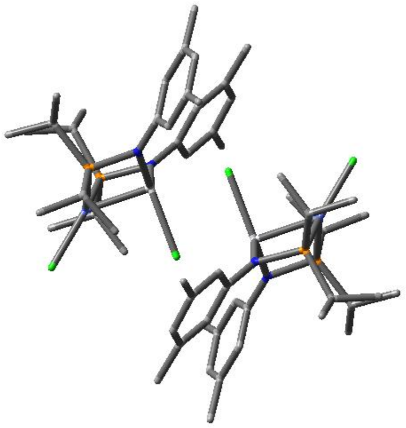

| Symbol | X | Y | Z |
|---|---|---|---|
| Co | -3.36312 | 0.02413 | 1.84609 |
| Ti | -1.91422 | 0.04479 | 0.14266 |
| Cl | -0.01924 | 0.10788 | 1.62451 |
| P | -3.70259 | -1.87826 | 0.71057 |
| P | -3.76494 | 1.8971 | 0.66773 |
| N | -2.65225 | -1.6243 | -0.61419 |
| N | -2.54982 | 1.78374 | -0.53416 |
| C | -2.5252 | -2.42003 | -1.79364 |
| C | -3.02679 | -1.93026 | -3.02782 |
| C | -2.91603 | -2.69084 | -4.21898 |
| C | -3.41442 | -2.13013 | -5.54437 |
| C | -2.31025 | -3.97082 | -4.15545 |
| C | -1.80302 | -4.48773 | -2.93377 |
| C | -1.16571 | -5.87093 | -2.8884 |
| C | -3.16391 | -3.44993 | 1.59799 |
| C | -1.68807 | -3.3008 | 2.04497 |
| C | -4.06642 | -3.76274 | 2.82317 |
| C | -5.44027 | -2.17682 | 0.02975 |
| C | -5.61055 | -3.52053 | -0.72862 |
| C | -6.53831 | -2.01265 | 1.12022 |
| C | -2.2958 | 2.64249 | -1.64609 |
| C | -2.04326 | 4.03221 | -1.47953 |
| C | -1.795 | 4.86864 | -2.59648 |
| C | -1.53579 | 6.3574 | -2.39678 |
| C | -1.77835 | 4.29496 | -3.89325 |
| C | -1.9954 | 2.90481 | -4.08498 |
| C | -1.90821 | 2.28637 | -5.47338 |
| C | -2.26694 | 2.09476 | -2.95683 |
| C | -5.40326 | 2.08394 | -0.26094 |
| C | -5.60337 | 3.4612 | -0.94703 |
| C | -6.63283 | 1.71679 | 0.61249 |
| C | -3.49662 | 3.46003 | 1.6857 |

| | | | |
|---|---|---|---|
| C | -2.05786 | 3.45115 | 2.26305 |
| C | -4.54108 | 3.58029 | 2.82921 |
| C | -1.90621 | -3.70033 | -1.76176 |
| H | -3.5156 | -0.94832 | -3.04469 |
| H | -3.72419 | -2.93515 | -6.23526 |
| H | -4.27509 | -1.45161 | -5.39993 |
| H | -2.61563 | -1.55116 | -6.04925 |
| H | -2.22472 | -4.57216 | -5.07102 |
| H | -0.66528 | -6.05251 | -1.92076 |
| H | -1.92493 | -6.66564 | -3.02841 |
| H | -0.41416 | -5.99436 | -3.69101 |
| H | -3.24434 | -4.2765 | 0.85998 |
| H | -1.00967 | -3.07248 | 1.20469 |
| H | -1.34268 | -4.24132 | 2.51534 |
| H | -1.58377 | -2.49066 | 2.78916 |
| H | -5.09487 | -4.04356 | 2.53562 |
| H | -3.63333 | -4.61308 | 3.38496 |
| H | -4.11397 | -2.89595 | 3.50823 |
| H | -5.53901 | -1.34439 | -0.69852 |
| H | -4.88664 | -3.63168 | -1.55264 |
| H | -5.49666 | -4.38273 | -0.04466 |
| H | -6.63067 | -3.57381 | -1.15867 |
| H | -6.30049 | -1.21018 | 1.84144 |
| H | -6.67092 | -2.94671 | 1.6945 |
| H | -7.50905 | -1.77515 | 0.64412 |
| H | -2.02607 | 4.4622 | -0.47183 |
| H | -1.43797 | 6.88138 | -3.36395 |
| H | -0.60225 | 6.52962 | -1.82568 |
| H | -2.35679 | 6.83916 | -1.83175 |
| H | -1.5821 | 4.93461 | -4.76424 |
| H | -2.6386 | 1.46591 | -5.59772 |
| H | -0.90121 | 1.85705 | -5.6442 |
| H | -2.09127 | 3.03526 | -6.26499 |
| H | -2.4433 | 1.02074 | -3.084 |
| H | -5.28777 | 1.30998 | -1.04939 |
| H | -4.77087 | 3.71908 | -1.62192 |
| H | -6.53645 | 3.44094 | -1.54477 |
| H | -5.71077 | 4.26955 | -0.19891 |
| H | -6.46172 | 0.8019 | 1.20382 |
| H | -7.51575 | 1.55111 | -0.03554 |
| H | -6.88652 | 2.53098 | 1.31508 |
| H | -3.61584 | 4.32105 | 0.99519 |
| H | -1.28921 | 3.32313 | 1.48084 |
| H | -1.93481 | 2.62854 | 2.99078 |

| | | | |
|---|---|---|---|
| H | -1.86438 | 4.40609 | 2.78945 |
| H | -5.56902 | 3.72792 | 2.45235 |
| H | -4.52473 | 2.68216 | 3.47423 |
| H | -4.28822 | 4.45379 | 3.4614 |
| H | -1.49386 | -4.07253 | -0.81666 |
| Co | 3.36239 | -0.02539 | -1.84631 |
| Ti | 1.91392 | -0.04521 | -0.14279 |
| Cl | 0.01893 | -0.10678 | -1.62489 |
| P | 3.70351 | 1.87678 | -0.71079 |
| P | 3.76411 | -1.89849 | -0.66795 |
| N | 2.65244 | 1.62382 | 0.61357 |
| N | 2.5503 | -1.78379 | 0.53499 |
| C | 2.52483 | 2.42048 | 1.79232 |
| C | 3.02694 | 1.93233 | 3.02693 |
| C | 2.91582 | 2.69407 | 4.21732 |
| C | 3.41482 | 2.13515 | 5.54323 |
| C | 2.30916 | 3.97358 | 4.15251 |
| C | 1.80138 | 4.48887 | 2.93038 |
| C | 1.1632 | 5.87162 | 2.88368 |
| C | 3.16688 | 3.44888 | -1.59866 |
| C | 1.69201 | 3.29999 | -2.049 |
| C | 4.07218 | 3.76215 | -2.82165 |
| C | 5.44092 | 2.17433 | -0.02886 |
| C | 5.61137 | 3.51791 | 0.72971 |
| C | 6.53943 | 2.01 | -1.11885 |
| C | 2.29683 | -2.64135 | 1.64796 |
| C | 2.04688 | -4.03177 | 1.4834 |
| C | 1.79902 | -4.86688 | 2.60142 |
| C | 1.54318 | -6.35651 | 2.40382 |
| C | 1.77998 | -4.29118 | 3.89727 |
| C | 1.99455 | -2.90038 | 4.087 |
| C | 1.9049 | -2.27984 | 5.47431 |
| C | 2.26591 | -2.09165 | 2.95786 |
| C | 5.40333 | -2.0857 | 0.25897 |
| C | 5.60304 | -3.46229 | 0.94655 |
| C | 6.63198 | -1.72117 | -0.61675 |
| C | 3.49422 | -3.46177 | -1.68502 |
| C | 2.05427 | -3.4535 | -2.25936 |
| C | 4.53649 | -3.58198 | -2.83049 |
| C | 1.9049 | 3.70029 | 1.75918 |
| H | 3.51656 | 0.9508 | 3.04477 |
| H | 3.7258 | 2.94106 | 6.23254 |
| H | 4.27482 | 1.4557 | 5.39922 |
| H | 2.61596 | 1.55768 | 6.04972 |

| | | | |
|---|---|---|---|
| H | 2.22338 | 4.57584 | 5.06745 |
| H | 0.66284 | 6.05204 | 1.91579 |
| H | 1.92191 | 6.66692 | 3.02314 |
| H | 0.41144 | 5.9953 | 3.68603 |
| H | 3.24572 | 4.27506 | -0.86004 |
| H | 1.01186 | 3.07034 | -1.21048 |
| H | 1.34739 | 4.2411 | -2.51876 |
| H | 1.58962 | 2.49085 | -2.79454 |
| H | 5.09967 | 4.0439 | -2.53156 |
| H | 3.6398 | 4.61201 | -3.38472 |
| H | 4.12215 | 2.8953 | -3.50645 |
| H | 5.53885 | 1.34186 | 0.69946 |
| H | 4.88747 | 3.62906 | 1.55372 |
| H | 5.49761 | 4.38023 | 0.04587 |
| H | 6.63149 | 3.571 | 1.1598 |
| H | 6.30093 | 1.20865 | -1.8411 |
| H | 6.67343 | 2.94453 | -1.69202 |
| H | 7.50964 | 1.77077 | -0.64256 |
| H | 2.03143 | -4.46345 | 0.47639 |
| H | 1.44045 | -6.87828 | 3.37168 |
| H | 0.61339 | -6.53158 | 1.82754 |
| H | 2.36833 | -6.8384 | 1.84493 |
| H | 1.5839 | -4.92979 | 4.76905 |
| H | 2.63339 | -1.45756 | 5.59784 |
| H | 0.89684 | -1.85241 | 5.64361 |
| H | 2.08896 | -3.027 | 6.26731 |
| H | 2.44044 | -1.01718 | 3.08362 |
| H | 5.28954 | -1.31066 | 1.0466 |
| H | 4.77169 | -3.71801 | 1.62367 |
| H | 6.53751 | -3.44252 | 1.54215 |
| H | 5.70763 | -4.2719 | 0.19936 |
| H | 6.46022 | -0.80805 | -1.21059 |
| H | 7.51564 | -1.55366 | 0.02982 |
| H | 6.88483 | -2.53742 | -1.31726 |
| H | 3.61504 | -4.32265 | -0.9946 |
| H | 1.28732 | -3.3262 | -1.47536 |
| H | 1.92911 | -2.63077 | -2.98659 |
| H | 1.86017 | -4.4084 | -2.78561 |
| H | 5.56535 | -3.72804 | -2.4555 |
| H | 4.51788 | -2.68451 | -3.47636 |
| H | 4.28338 | -4.45639 | -3.46131 |
| H | 1.49209 | 4.0711 | 0.81374 |
| Cl | 3.33642 | -0.00016 | -4.03229 |
| Cl | -3.33932 | -0.00048 | 4.03217 |

## References

1. Zhou, W.; Saper, N. I.; Krogman, J. P.; Foxman, B. M.; Thomas, C. M. *Dalton Trans.* **2014**, *43*, 1984.
2. Greenwood, B. P; Forman, S. I.; Rowe, G. T.; Chen, C.-H.; Foxman, B. M.; Thomas, C. M. *Inorg. Chem.* **2009**, *48*, 6251.
3. Apex2, Version 2 User Manual, M86-E01078, Bruker Analytical X-ray Systems, Madison, WI, June 2006.
4. Frisch, M. J.; Trucks, G. W.; Schlegel, H. B.; Scuseria, G. E.; Robb, M. A.; Cheeseman, J. R.; Scalmani, G.; Barone, V.; Mennucci, B.; Petersson, G. A.; Nakatsuji, H.; Caricato, M.; Li, X.; Hratchian, H. P.; Izmaylov, A. F.; Bloino, J.; Zheng, G.; Sonnenberg, J. L.; Hada, M.; Ehara, M.; Toyota, K.; Fukuda, R.; Hasegawa, J.; Ishida, M.; Nakajima, T.; Honda, Y.; Kitao, O.; Nakai, H.; Vreven, T.; Montgomery Jr., J. A.; Peralta, J. E.; Ogliaro, F.; Bearpark, M.; Heyd, J. J.; Brothers, E.; Kudin, K. N.; Staroverov, V. N.; Kobayashi, R.; Normand, J.; Raghavachari, K.; Rendell, A.; Burant, J. C.; Iyengar, S. S.; Tomasi, J.; Cossi, M.; Rega, N.; Millam, J. M.; Klene, M.; Knox, J. E.; Cross, J. B.; Bakken, V.; Adamo, C.; Jaramillo, J.; Gomperts, R.; Stratmann, R. E.; Yazyev, O.; Austin, A. J.; Cammi, R.; Pomelli, C.; Ochterski, J. W.; Martin, R. L.; Morokuma, K.; Zakrzewski, V. G.; Voth, G. A.; Salvador, P.; Dannenberg, J. J.; Dapprich, S.; Daniels, A. D.; Farkas, O.; Foresman, J. B.; Ortiz, J. V.; Cioslowski, J.; Fox, D. J., Gaussian, Inc., Wallingford, CT, 2009.
5. Becke, A. D. *Phys. Rev. A.***1988,** *38*, 3098.
6. Perdew, J. P. *Phys. Rev. B.***1986**, *33*, 8822.
7. (a) Hay, P. J.; Wadt, W. R. *J.Chem. Phys.***1985**, *82*, 299. (b) Hay, P. J.; Wadt, W. R. *J. Chem. Phys.***1985**, *82*, 270.
8. Dunning, T. H.; Hay, P. J. *Modern Theoretical Chemistry. Vol. 3*, Plenum,New York, **1976**.
9. Glendening, E. D.; Reed, A. E.; Carpenter, J. E.; Weinhold, F. *NBO Version 3.1.*
10. Palatinus, L.; Chapuis, G.; *J. Appl. Cryst.* **2007**, 40, 786.
11. a) Betteridge, P. W.; Carruthers, J. R.; Cooper, R. I.; Prout, K.; Watkin, D. J. *J. Appl. Cryst.***2003**, *36*, 1487; b) Prout, C.K;. Pearce,L.J. CAMERON, Chemical Crystallography Laboratory, Oxford, UK, 1996.
12. Cooper, R. I.; Thompson, A. L.; Watkin, D. J. *J. Appl. Cryst.***2010**, *43*, 1100.
13. Altomare, A; Cascarano, G; Giacovazzo, G.; Guagliardi, A.; Burla, M. C.; Polidori,G.;Camalli, M. *J. Appl. Cryst.* **1994**, *27*, 435.
14. Cooper, R. I.; Gould, R. O.; Parsons, S.; Watkin, D. J. *J. Appl. Cryst.***2002**, *35*,168.
15. Donnay,G.; Donnay, J. D. H. *Canadian Mineralogist*, **1974**,*12*, 422.
